# Sclerostin Depletion Induces Inflammation in the Bone Marrow of Mice

**DOI:** 10.3390/ijms22179111

**Published:** 2021-08-24

**Authors:** Cristine Donham, Betsabel Chicana, Alexander G. Robling, Asmaa Mohamed, Sonny Elizaldi, Michael Chi, Brian Freeman, Alberto Millan, Deepa K. Murugesh, Nicholas R. Hum, Aimy Sebastian, Gabriela G. Loots, Jennifer O. Manilay

**Affiliations:** 1 Department of Molecular and Cell Biology, School of Natural Sciences, University of California, Merced, 5200 North Lake Road, Merced, CA 95343, USA; cdonham@ucmerced.edu (C.D.); bchicanaromero@ucmerced.edu (B.C.); amohamed4@ucmerced.edu (A.M.); selizaldi@ucmerced.edu (S.E.); mchi3@ucmerced.edu (M.C.); bfreeman3@ucmerced.edu (B.F.); amillan-hernandez@ucmerced.edu (A.M.); loots1@llnl.gov (G.G.L.); 2 Quantitative and Systems Biology Graduate Program, University of California, Merced, 5200 North Lake Road, Merced, CA 95343, USA; hum3@llnl.gov; 3 Department of Anatomy, Cell Biology, and Physiology, Indiana University School of Medicine, Indianapolis, IN 46202, USA; arobling@iupui.edu; 4 Roudebush VA Medical Center, Indianapolis, IN 46202, USA; 5 Physical and Life Sciences Directorate, Lawrence Livermore National Laboratory, Livermore, CA 94550, USA; murugesh2@llnl.gov (D.K.M.); sebastian4@llnl.gov (A.S.)

**Keywords:** osteoimmunology, osteopetrosis, genetic animal models, aging

## Abstract

Romosozumab, a humanized monoclonal antibody specific for sclerostin (SOST), has been approved for treatment of postmenopausal women with osteoporosis at a high risk for fracture. Previous work in sclerostin global knockout (*Sost*^−/−^) mice indicated alterations in immune cell development in the bone marrow (BM), which could be a possible side effect in romosozumab-treated patients. Here, we examined the effects of short-term sclerostin depletion in the BM on hematopoiesis in young mice receiving sclerostin antibody (Scl-Ab) treatment for 6 weeks, and the effects of long-term *Sost* deficiency on wild-type (WT) long-term hematopoietic stem cells transplanted into older cohorts of *Sost*^−/−^ mice. Our analyses revealed an increased frequency of granulocytes in the BM of Scl-Ab-treated mice and WT→*Sost*^−/−^ chimeras, indicating myeloid-biased differentiation in *Sost*-deficient BM microenvironments. This myeloid bias extended to extramedullary hematopoiesis in the spleen and was correlated with an increase in inflammatory cytokines TNFα, IL-1α, and MCP-1 in *Sost*^−/−^ BM serum. Additionally, we observed alterations in erythrocyte differentiation in the BM and spleen of *Sost*^−/−^ mice. Taken together, our current study indicates novel roles for *Sost* in the regulation of myelopoiesis and control of inflammation in the BM.

## 1. Introduction

Two rare human bone disorders, sclerosteosis and van Buchem disease, are characterized by dramatic increases in bone mineral density (BMD) and have been genetically traced to mutations in the *Sost* gene locus, which codes for sclerostin [1,2]. Sclerostin protein is predominantly secreted by osteocytes and functions as a negative regulator of bone formation. Through the binding of low-density lipoprotein-related protein 5 and 6 (LRP5 and LRP6), SOST has been shown to inhibit canonical Wnt (wingless-related integration site) signaling [3]. LRP4 is also required for Wnt inhibition by sclerostin [4]. Additionally, when Wnt is inhibited, osteoprotegerin (OPG) is decreased, and there is an increase in bone resorption [5]. In *Sost*^−/−^ mice where Wnt is upregulated, OPG is concurrently increased [5]. This demonstrates that *Sost* not only regulates bone formation, it also regulates bone resorption. The increase in BMD in *Sost*^−/−^ mice and in humans with decreased SOST function has led to consideration of sclerostin as a potential target for treatment of osteoporosis and other diseases associated with low bone mineral density. A humanized monoclonal antibody against sclerostin, termed romosozumab (brand name EVENITY^®^), has been approved in the U.S. for women with osteoporosis after menopause at a high risk for fracture. In several phase III clinical trials, romosozumab was shown to decrease the risk of vertebral fractures up to 73% and increase total hip area BMD by 3.2% [6].

Hematopoiesis occurs mainly in the bone marrow (BM). There is increasing evidence on the effects of altered bone homeostasis on hematopoiesis [7,8]. Self-renewal and quiescence of long-term hematopoietic stem cells (LT-HSCs) and hematopoietic differentiation involves many cell signaling pathways, including Wnt, Notch, FGF, and Hedgehog [9]. Several studies regarding Wnt signaling and hematopoiesis have produced contradictory results [10,11,12]. Instead, there have been a few reconciling propositions, including the idea that canonical Wnt signaling regulates hematopoiesis in a dosage-dependent fashion, Wnt signaling differentially affects fetal and adult hematopoietic stem cells (HSCs), and interactions between canonical and noncanonical Wnt pathways influence the interpretation of these results [13].

Under homeostatic conditions, LT-HSCs maintain a quiescent state in the BM, providing a lifelong reservoir of multipotent cells that replenish hematopoietic populations as they are depleted by use or age. Since the discovery of HSCs in 1961 [14], a growing list of hematopoietic stem and progenitor cell (HSPC) populations have been described, along with a set of cell surface markers to define them. Here, unless otherwise specified, we define the conglomerate HSPCs as LSKs (Lin^−^, Sca1^+^, cKit^+^), and LT-HSCs as LSK, CD150^+^, CD48^−^, Flk2^−^ [15,16,17]. In an adult mouse, LT-HSCs give rise to all mature blood lineages. As cells progress from LT-HSCs to short-term hematopoietic stem cells (ST-HSCs) and then to multipotent progenitors 2, 3, or 4 (MPP2, MPP3, or MPP4), cells are generally thought to split into two different paths, the myeloid and lymphoid pathways. It has been demonstrated that MPP2 and MPP3 contribute largely to myeloid cells, and MPP4 to lymphoid cells [18]. Lymphopoiesis produces cell types, such as mature B, T, and natural killer (NK) cells, whereas myelopoiesis produces a larger variety of mature cells, such as monocytes, granulocytes, and erythrocytes.

Organismal aging is characterized by increased inflammation and decreased HSC functional capacity. This general decline in HSC function includes a reduction in long-term repopulating potential, homing, and engraftment after transplantation [19], a decrease in lymphopoiesis, and increase in myelopoiesis [20,21], as well as an increase in the total number of LT-HSCs [22]. Aging is also associated with a chronic inflammatory phenotype that has been characterized by anemia, immunosenescense, and thrombocytosis [23], as well as overproduction of the inflammatory cytokines interleukin-1 (IL-1), tumor necrosis factorα (TNFα), and interleukin-6 (IL-6) [24].

In 1978, the concept of a hematopoietic stem cell niche was introduced, and is still being explored today [25]. The spatial localization of HSC niches in the BM remains controversial, with early work suggesting that hematopoiesis is maintained in homogenously distributed niches, while more recent work suggests distinct niche structures [26]. However, it is agreed upon that local BM microenvironments maintain HSCs and regulate their function by producing factors that act directly on HSCs, such as stem cell factor (SCF), chemokine (C-X-C motif) 12 (CXCL12), and thrombopoietin. SCF binds to the KIT receptor on HSCs and is required for HSC maintenance [27]. CXCL12 is involved in HSC maintenance and retention in the BM through activation signaling of C-X-C motif chemokine receptor 4 (CXCR4) on HSCs [28]. CXCL12–CXCR4 signaling also regulates several myeloid and lymphoid progenitors’ proliferation and retention in the BM [29,30]. Thrombopoietin activates signaling through myeloproliferative leukemia protein on HSCs and is required for HSC maintenance [31]. Thrombopoietin is also important in megakaryocyte and platelet production [32].

Our previous work demonstrated that *Sost* deficiency in mice affects B lymphocyte development in a non-cell autonomous manner, CXCL12 levels are diminished in the bones of *Sost*^−/−^ mice, but Wnt signaling in B lymphocytes does not appear to be affected [33]. In this study, we extended our investigation to focus on HSC function and fate in sclerostin-deficient BM microenvironments. Specifically, we hypothesized that removal of sclerostin would influence LT-HSC self-renewal, maintenance, and differentiation.

## 2. Results

### 2.1. Sclerostin-Depleting Antibody Treatment Changes Hematopoietic Differentiation

**Figure 1:**
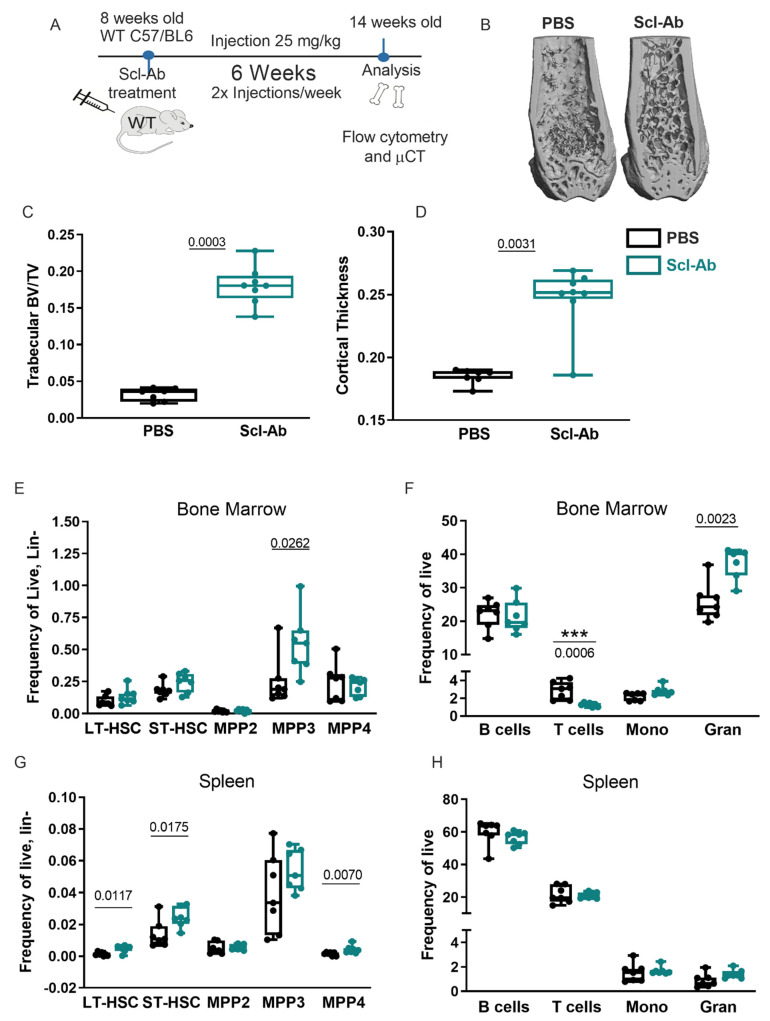
Sclerostin-depleting antibodies change hematopoietic differentiation. (**A**) Experimental scheme for Scl-Ab treatment and analysis; (**B**) micro-CT images of femurs from PBS and Scl-Ab-treated mice; (**C**) trabecular BV/TV; (**D**) cortical bone mineral density; (**E**) bone marrow frequency of LSK progenitors; (**F**) bone marrow frequency of mature lineages; (**G**) splenic frequency of LSK progenitors; (**H**) splenic frequency of mature lineages. Female mice of 8 weeks of age were used for this study (*n* = 7 for each group). The actual *p*-values are shown, underlined.

*Sost*^−/−^ mice display a drastic reduction in the BM cavity volume [34], which we hypothesized affects the hematopoietic stem cell niches within the BM [35,36]. Given the known relationship between Wnt signaling on hematopoietic stem cell self-renewal and function [37], and that sclerostin is a Wnt signaling antagonist [3,38], we hypothesized that removal of sclerostin through administration of neutralizing antibodies would promote HSC self-renewal. To test if acute depletion of *SOST* changed hematopoiesis, we performed studies in which *SOST* was depleted using sclerostin-specific antibodies (Scl-Ab), administered subcutaneously in 8-week-old mice in multiple doses for 6 weeks (Figure 1A). Scl-Ab treatment resulted in increased trabecular volume/total volume (BV/TV) and midshaft cortical thickness, similar to that observed in the *Sost*^−/−^ mice (Figure 1B–D) [34,39]. However, the BM total cellularity was unchanged (data not shown). Analysis of B cell development in the BM of Scl-Ab-treated mice showed an increase in B cell progenitors (Hardy Fractions B–D), followed by a decrease in the mature recirculating B cells (Hardy Fraction F) (Figure S1E), similar to the altered B cell development observed in the BM of 12–15-week-old *Sost*^−/−^ mice [33]. In the spleens of the Scl-Ab-treated mice, a significant decrease in mature B cell populations (CD19^+^B220^+^ and IgM^+^B220^+^) was observed (Figure S1F).

The frequency of the HSPC subpopulations (LT-HSC, ST-HSC, MPP2, MPP3, and MPP4) was unaffected in the BM of Scl-Ab-treated mice, with the exception of a slight increase in the frequency of MPP3 (a myeloid-biased progenitor) (Figure 1E). This change in frequency, however, did not result in a difference in MPP3 cell numbers (Figure S1A). In the BM, CD3^+^ T lymphocytes were reduced and CD11b^+^ Gr1^+^ granulocytes were increased in both frequency and absolute number after Scl-Ab treatment (Figure 1F and Figure S1B), consistent with the increase in MPP3 frequency. To test if these hematopoietic changes were confined to the bone, we also examined HSPC and hematopoietic lineages in the spleen. In contrast to the BM, the spleens displayed an increase in LT-HSCs, ST-HSCs, and MPP4s (a lymphoid-biased progenitor) in frequency and cellularity (Figure 1G and Figure S1C). In the spleen, there was no significant change in granulocyte frequency, but an increase in granulocyte cell number was observed in Scl-Ab-treated mice (Figure 1H and Figure S1D).

### 2.2. Lack of Sclerostin in the Bone Does Not Affect Hematopoietic Progenitor Distributions in the Bone Marrow

Our observation that acute Scl-Ab treatment increased MPP3 distribution in the BM and LT-HSCs, ST-HSCs, and MPP4s in the spleen led us to inquire whether similar alterations would also be observed longer-term in a chronic *Sost* deficiency model. We elected to use a transplantation model, as subtle changes in hematopoiesis in response to the bone microenvironment (Figure S2) can be enhanced using transplantation approaches.

**Figure 2:**
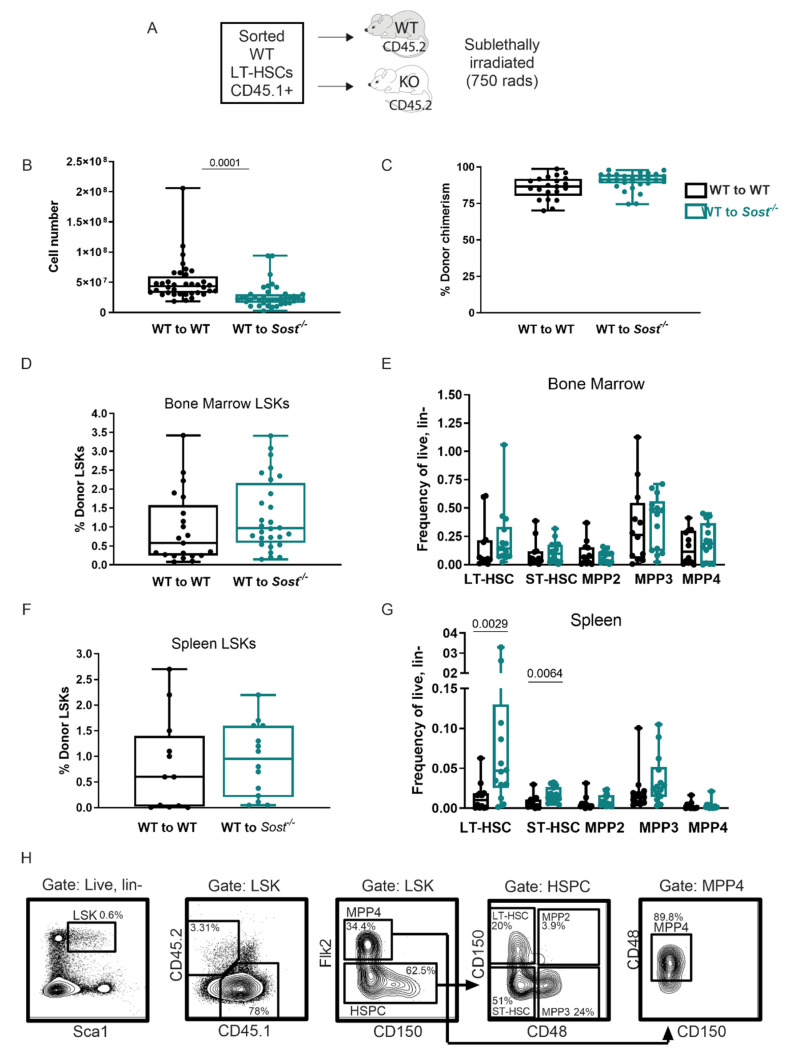
Lack of sclerostin in the bone does not alter hematopoietic progenitor distribution in the bone marrow. (**A**) Experimental scheme of LT-HSC transplant model to study hematopoiesis long-term in *Sost*-deficient BM microenvironments; (**B**) total bone marrow cellularity in WT→WT (control) and WT→*Sost*^−/−^ chimeras; (**C**) percent donor chimerism; (**D**) frequency of donor-derived BM LSKs; (**E**) frequency of donor-derived BM HSPCs; (**F**) frequency of donor-derived splenic LSKs; (**G**) frequency of donor-derived spleen HSPCs; (**H**) representative FACS plots depicting gating strategy for HSPC gating in chimeras. Ages of the BM donor mice ranged from 9–17 weeks. Ages of the recipients ranged from 16–22 weeks, when the phenotype of the *Sost*^−/−^ BM is already established. Both male and female donors and hosts were used. Donor LT-HSC cell numbers ranged from 235–400 cells per mouse. Sort purities ranged from 90.3–100%. Analysis of BM and spleens was performed 19–29 weeks post-transplantation. Data shown are compiled from 4 independent experiments. The actual *p*-values are shown, underlined.

Equal numbers of purified wildtype (WT) CD45.1^+^ LT-HSCs (LSK, CD150^+^ CD48^−^) cells were transplanted into sub-lethally irradiated (750 rads) congenic CD45.2^+^ WT or *Sost*^−/−^ hosts (Figure 2A). The use of the congenic CD45.1 and CD45.2 mice is an established approach that permits the tracking of donor-derived (CD45.1^+^) hematopoiesis separate from host-derived (CD45.2^+^) hematopoiesis after transplant. This model also allows for comparison of fully developed LT-HSCs isolated from the same source of adult WT mice, and their engraftment and differentiation after transfer into *Sost*^−/−^ or control microenvironments. Sixteen weeks after LT-HSC transplantation, the recipients were analyzed for BM and spleen cellularity, donor hematopoietic chimerism, and the frequencies of donor-derived hematopoietic progenitors. As expected, total bone marrow cellularity was significantly decreased in the *Sost*^−/−^ recipients (Figure 2B [33]). Donor percent hematopoietic chimerism within the BM was achieved at similar levels in WT and *Sost*^−/−^ recipients (Figure 2C), indicating no effect of the *Sost*^−/−^ microenvironment on donor WT LT-HSC engraftment after transplantation. Additionally, the frequency of donor-derived LSKs was unchanged in the BM (Figure 2D). We further analyzed the types of HSPCs in the chimeras for LT-HSCs, ST-HSCs, MPP2, MPP3, and MPP4 subpopulations (Figure 2E,G). Within the BM of WT→*Sost*^−/−^ chimeras, no statistically significant changes in proportions or absolute numbers of any HSPC subpopulations were observed Figure 2E and Figure S3A). Furthermore, no change in the expression of self-renewal and cell cycle genes *p21Cip1* and *HoxB4* were observed in *Sost*^−/−^ LT-HSCs sorted from the BM (Figure S3C, [40]), and no evidence of changes in LSK quiescence or enhanced cell cycling after 5-fluorouracil treatment was observed in *Sost*^−/−^ mice (Figure S3D–G). In the BM of non-transplanted *Sost*^−/−^ mice, we observed a decrease in early apoptotic LSKs, but no change in Ki67^+^ proliferating LSKs (Figure 5A,C,E). In the spleen of non-transplanted *Sost*^−/−^ mice, we observed an increase in early apoptotic and live LSKs, with a decrease in late apoptotic LSKs (Figure 5A,D). However, similar to Scl-Ab-treated mice, in LT-HSC and ST-HSC, frequencies and absolute numbers in the spleen were increased in the WT→ *Sost*^−/−^ chimeras (Figure 2G and Figure S3B). In addition, absolute numbers of MPP2 and MPP3 in the spleen were also increased (Figure S3B). No difference in hematopoiesis was observed in reciprocal *Sost*^−/−^→WT chimeras ([33] and Figure S4).

### 2.3. Lack of Sclerostin in the Bone Microenvironment Results in a Myeloid Bias

**Figure 3:**
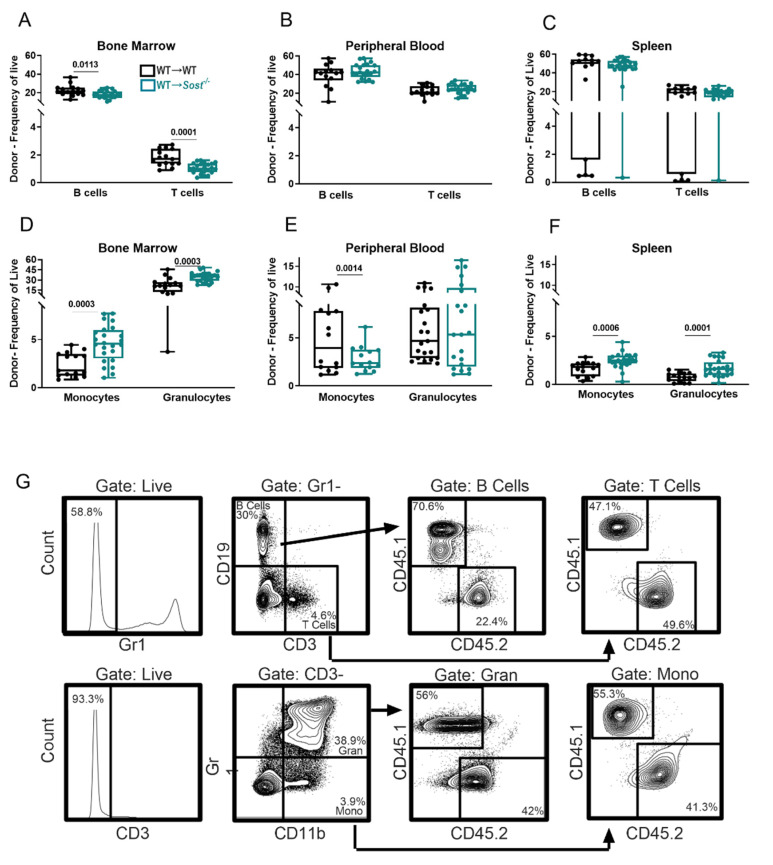
Lack of sclerostin in the bone microenvironment results in a myeloid bias. Frequencies of donor-derived lineage populations in BM (**A**), peripheral blood (**B)**, and spleen (**C**) in chimeras; cellularity of donor-derived lineage populations in BM (**D**), peripheral blood (**E**), and spleen (**F**) in chimeras; (**G**) representative FACS plots depicting gating strategy for mature lineages in chimeras. Data shown are from a WT→*Sost*^−/−^ chimera. Data are from the same experiments described in Figure 2. The actual *p*-values are shown, underlined.

We also analyzed hematopoietic differentiation broadly in the chimeras, using an antibody cocktail to quantify CD19^+^ B lymphocytes, CD3^+^ T lymphocytes, CD11b^+^ Gr1^−^ monocytes, and CD11b^+^ Gr1^+^ granulocytes (Figure 3). In the BM, the frequency and number of donor-derived T cells and B cells were decreased in WT→*Sost*^−/−^ chimeras compared to WT→WT controls (Figure 3A and data not shown). In addition, a significant increase in the frequency of donor-derived monocytes and granulocytes was observed in the BM of *Sost*^−/−^ hosts (Figure 3D), but with decreased absolute numbers compared to controls (data not shown). MPP2, MPP3, and MPP4 HSPC progenitor frequencies and absolute numbers were unchanged (Figure 2E and Figure S3). In the spleens of WT→*Sost*^−/−^ chimeras, we observed an increase in the number of splenic MPP2 and MPP3 cells (Figure S3B), accompanied with increased monocyte and granulocyte frequencies (Figure 3D) and granulocyte and T cell cellularity in the WT→*Sost*^−/−^ spleens (data not shown). The peripheral blood of the WT→*Sost*^−/−^ showed a decreased monocyte frequency (Figure 3E) but was similar to controls in B cells, T cells, and granulocytes (Figure 3B,E).

**Figure 4:**
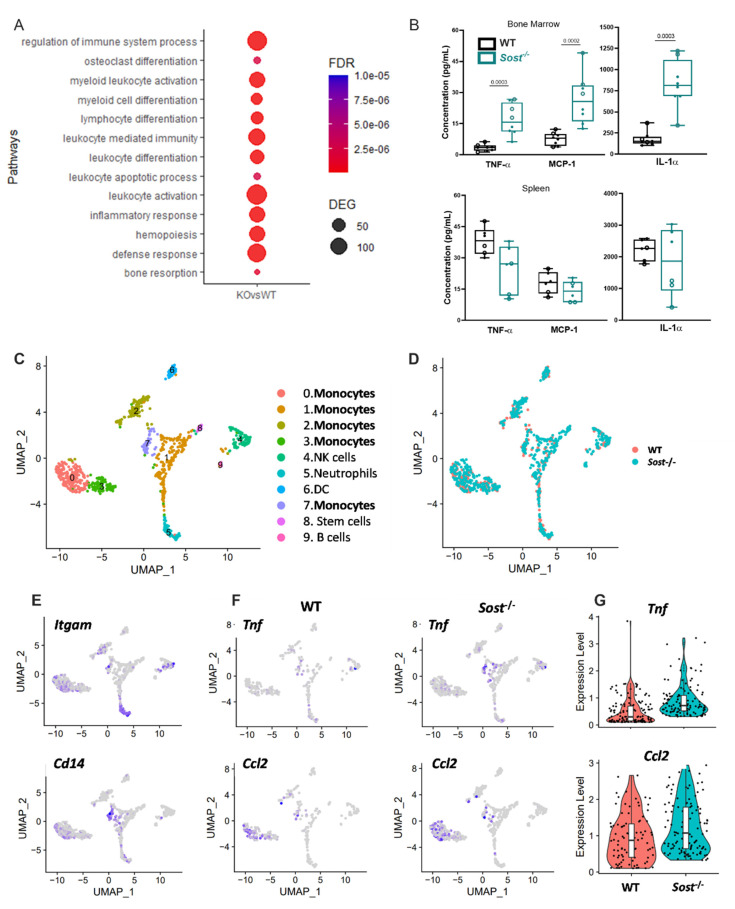
Elevated levels of inflammatory cytokines in *Sost*^−/−^ bone marrow. (**A**) Dot plot showing enriched gene ontology categories in bulk RNA-seq analysis of LT-HSCs sorted from WT→Sost^−/−^ bone marrow chimeras compared to WT→WT chimeras; (**B**) TNFα, MCP-1, and IL-1α concentrations in WT and *Sost*^−/−^ bone marrow (top) and spleen (bottom). Open circles represent male mice and filled circles represent female mice. Male (4 WT and 4 *Sost*^−/−^) and female (4 WT and 4 *Sost*^−/−^) mice ranging in ages of 25–36 weeks were used. All samples were analyzed using a single LEGENDPLEX kit. The actual *p*-values are shown, underlined. (**C**) UMAP plots showing immune cell clusters in scRNA-seq analysis of residual bone marrow cells remaining after depletion of CD45^+^ cells and collagenase digestion of WT and *Sost^−/^*^−^ bones. Cell clusters are colored based on cluster identity; (**D**) UMAP plots with cells colored based on genotype; (**E**) feature plot showing the expression of *Itgam* (*Cd11b*) and *Cd14* in various immune clusters; (**F**) feature plot showing the expression of *Tnf* and *Ccl2* in immune cells from WT (left) and *Sost*^−/−^ bones (right). (**G**) Violin plots of expression levels on all cells expressing *Tnf* and *Ccl2*. ScRNA-seq data are from one experiment, with two 10-week-old mice per genotype.

Our observations of increased frequency of monocytes and granulocytes in the BM and spleen are consistent with skewing of LT-HSC hematopoietic differentiation towards the myeloid lineages in the *Sost*^−/−^ BM microenvironment. Our observation of no changes in myeloid lineage frequencies or absolute numbers in reciprocal *Sost*^−/−^→WT chimeras and in competitive LSK transplantation studies, where equal numbers of LSKs from WT B6.SJL and *Sost*^−/−^ mice were co-transplanted into WT recipients (data not shown), supports that the source of the myeloid bias is not cell-intrinsic to the hematopoietic stem cells or progenitors, and that the microenvironment of *Sost*^−/−^ mice is conducive for myeloid expansion. To investigate this at the molecular level, we sorted LT-HSCs from WT→ *Sost*^−/−^ chimeras and control WT→WT chimeras for bulk RNA-seq analysis. Gene ontology (GO) analysis of upregulated genes identified several enriched biological processes, including myeloid activation, myeloid differentiation, osteoclast differentiation, and inflammatory response pathways in LT-HSCs in the WT→ *Sost*^−/−^ chimeras (Figure 4A and Figure S5, Tables S1 and S2).

### 2.4. Sost^−/−^ Mice Express High Levels of Inflammatory Cytokines in the Bone Marrow

The bulk RNA-seq analysis indicated that LT-HSCs in the bone marrow of WT → *Sost*^−/−^ chimeras were experiencing an inflammatory microenvironment. The immune system is very responsive to inflammatory signaling caused by insults and environmental disturbances [41]. Pietras et al. [42] and others have shown that inflammatory responses in the bone marrow are accompanied by changes in the blood system, including overproduction of myeloid cells and decrease in production of lymphoid cells. We hypothesized that the *Sost*^−/−^ BM microenvironments may express increased levels of proinflammatory cytokines. We assessed 13 different inflammatory cytokines in the serum of the *Sost*^−/−^ BM and spleen, and found TNFα, monocyte chemoattractant protein-1 (MCP-1, gene name *Ccl2*), and IL-1α to be significantly increased in the BM (Figure 4B, top). This increase was localized to the *Sost*^−/−^ BM, as no difference in TNFα, MCP-1, and IL-1α was observed in the spleen (Figure 4B, bottom) or peripheral blood (data not shown). No differences in IL-1β, IL-6, IL-10, IL-12p70, IL-17A, IL-23, IL-27, interferon-β (IFN-β), IFN-γ, and granulocyte-macrophage colony-stimulating factor (GM-CSF) were observed in any tissue examined (data not shown).

### 2.5. Bone Marrow Monocytes in Sost^−/−^ Mice Exhibit Upregulated Tnf and Ccl2 Gene Expression

To identify the cellular source of TNFα, MCP-1, and IL-1α, we used single-cell RNA sequencing (scRNA-seq) of residual CD45^+^ (*Ptprc*) bone marrow cells from collagenase-digested WT and *Sost*^−/−^ bones. scRNA-seq revealed 10 distinct immune cell populations found in both WT and *Sost*^−/−^ mice (Figure 4C,D and Figure S6), including subsets of monocytes (Populations 0, 1, 2, 3, and 7) defined by expression of *Itgam1* (*Cd11b*) and *Cd14* (Figure 4E). Monocytes in Populations 3 and 7 showed ~2.25 times higher *Tnfa* expression on a per cell basis in the *Sost*^−/−^ bone (Figure 4F) than WT. *Ccl2* (MCP-1) was found equivalently on a per cell basis in monocytes in Populations 0 and 7, but there were more *Ccl2*^+^ cells in those subpopulations in *Sost*^−/−^ mice. *Ccl2* was also expressed in CD45-negative mesenchymal stromal cells and fibroblasts at equivalent levels in WT and *Sost*^−/−^ mice (data not shown). The higher expression levels of *Tnf* and *Ccl2* are shown in Figure 4G. *IL-1a* was not detected in any CD45^+^ or CD45^−^ cells in this analysis (data not shown).

### 2.6. Evidence of Extramedullary Hematopoiesis in Sost^−/−^ Mice

CXCL12 is a chemokine that can influence HSC retention in the BM [43], and SCF is important for HSC maintenance. To further investigate the effects of *Sost* on the bone microenvironment, we analyzed the expression of CXCL12 and SCF in mesenchymal stem cells (MSCs), osteoblasts (OBs), endothelial cells (ECs), and other CD45^−^ bone cells after collagenase-digestion of WT and *Sost*^−/−^ tibiae and femora. The distribution of MSCs, OBs, and ECs differed in the *Sost*^−/−^ mice compared to controls (Figure S7D) [44]. We observed a lower expression of *Cxcl12* in the whole *Sost*^−/−^ bone compared to controls, but this decreased expression could not be attributed to MSCs, OBs, or ECs (Figure S6B). In addition, *Scf* levels were similar in *Sost*^−/−^ and control bones (Figure S7C) [40].

**Figure 5:**
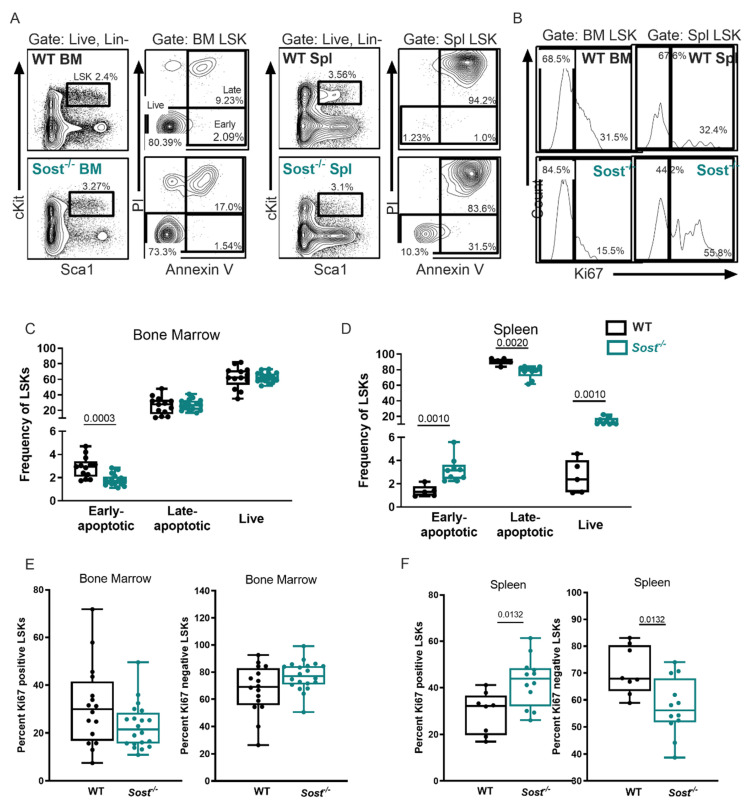
BM and splenic LSKs in Sost^−/−^ mice display opposite patterns of proliferation and early apoptosis. (**A**) Representative FACS analysis of LSKs stained with annexin V and PI, showing live cells, and cells in early and late apoptosis; (**B**) FACS analysis of LSKs stained with Ki67; (**C**) summary of apoptosis staining results in the LSKs of the BM; (**D**) summary of apoptosis staining results in the LSKs of the spleen; (**E**) frequencies of Ki67^+^ BM LSKs; (**F**) frequencies of Ki67^+^ splenic LSKs. Data on apoptosis were compiled from 2 independent experiments, using male mice with ages ranging from 6 to 63 weeks, with age-matched controls in the same experiment. Data on proliferation were compiled from 3 independent experiments, using male and female mice with ages ranging from 23 to 54 weeks. The actual *p*-values are shown, underlined.

Insults to the BM environment, such as inflammation [45,46,47,48], can result in extramedullary hematopoiesis (EMH) in the spleen. The increase in the splenic HSPC compartment indicated EMH was prominent in the spleens of WT→*Sost*^−/−^ chimeras. The reduced levels of CXCL12 in the *Sost*^−/−^ bone also suggested that HSCs may not be retained within the BM, and perhaps egress to the spleen. Consistent with this, a significant increase in the frequency and cellularity of splenic LT-HSC and ST-HSCs was evident (Figure 2G and Figure S3B). Splenic MPP2 and MPP3 populations were also increased in cellularity (Figure S3B). To determine the kinetics of the onset of EMH, we analyzed the LT-HSC frequency in the spleens of unmanipulated *Sost*^−/−^ mice as a function of age, and observed that LT-HSCs in *Sost*^−/−^ spleens were significantly increased by 34 weeks of age, whereas the increase in ST-HSCs began as early as 15 weeks (Figure S8D–G). The spleen contains an increase in proliferating LSK HSPCs in active cell cycle, as measured by Ki67 expression (Figure 5B,F). Paradoxically, an increase in early apoptotic LSKs was also observed in *Sost*^−/−^ spleens. The size of the spleens of *Sost*^−/−^ mice was visibly enlarged compared to controls (Figure S8A,C), and we observed a trend toward an increase in the splenic red pulp area, where LT-HSCs and erythrocytes are found during EMH [45] (Figure S8B,C). To test their hematopoietic function, we purified splenic HSPCs from *Sost*^−/−^ mice and transplanted them into sub-lethally irradiated WT hosts (Figure S9A). There was no difference in hematopoietic engraftment or differentiation amongst HSPCs isolated from WT BM, *Sost*^−/−^ BM, or *Sost*^−/−^ spleens (Figure S9D–G). Taken together, these data indicate EMH is elevated in the *Sost*^−/−^ mice, but that the microenvironment of the *Sost*^−/−^ spleen does not seem to permanently harm the engraftment or differentiation capability of HSPCs.

### 2.7. Erythrocyte Development Is Altered in Sost^−/−^ Mice

**Figure 6:**
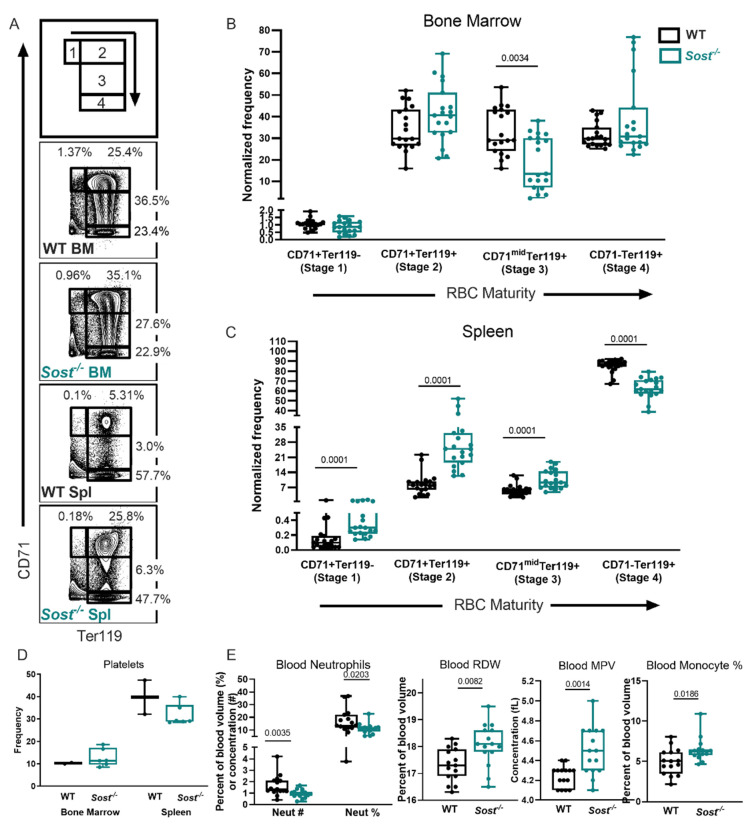
RBC development in the *Sost*^−/−^ BM is altered and developmentally blocked in the *Sost*^−/−^ spleen. (**A**) Representative FACS plots depicting stages of RBC development. (**B**) Normalized frequencies of stages 1–4 bone marrow RBC progenitor populations. (**C**) Normalized frequencies of stages 1–4 splenic RBC populations. (**D**) Bone marrow platelet frequency measured by flow cytometry. (**E**) CBC analysis depicting neutrophil concentration and cellularity, red blood cell distribution width (RDW), and mean platelet volume (MPV). Data on RBC development in the BM and spleen of mice were compiled from 3 independent experiments, and male and female mice of 9–60 weeks of age were used. Platelet analyses in the BM and spleen were compiled from 2 independent experiments, using mice of both sexes ranging in age from 6 to 60 weeks. Peripheral blood data were compiled from 2 independent experiments, using mice of both sexes ranging in age from 25 to 48 weeks. The actual *p*-values are shown, underlined.

Chronic inflammation has been shown to induce anemia [49], and we hypothesized the increase in TNFα, MCP-1, and IL-1α in the BM of *Sost*^−/−^ mice would result in a decrease of mature erythrocytes (red blood cells, RBC). To determine if mature RBCs and erythrocyte progenitors in the BM were reduced, we performed flow cytometry analysis with Ter119 and CD71 cellular markers, which allows for discrimination of developmentally distinct RBC progenitor populations (stage 1: CD71^+^Ter119^−^, stage 2: CD71^+^Ter119^+^, stage 3: CD71^mid^Ter119^+^, and stage 4: CD71^−^ Ter119^+^) [50]. The most immature progenitors (stage 1) express intermediate levels of Ter119 and high levels of CD71, and, as they mature, begin to downregulate CD71 and upregulate Ter119 (Figure 6A). In *Sost*^−/−^ BM we observed a decrease in stage 3 frequency (Figure 6B). Consistent with this, the frequency of 7AAD^−^ annexin V^−^ (live) stage 3 progenitors was significantly decreased (data not shown). Interestingly, in the spleen, we observed that stages 1–3 are increased, and stage 4 is decreased, indicating a possible RBC developmental block (Figure 6C). However, no difference in early or late apoptotic cells was observed at any stage of erythrocyte progenitors in the bone marrow or spleen (data not shown).

We utilized a Hemavet for complete blood cell analysis to further examine RBC differences in the *Sost*^−/−^ mice (Table S3). This analysis revealed an increase in red blood cell distribution width (RDW) in *Sost*^−/−^ mice (Figure 6E). A rise in RDW values indicates greater variation in RBC size and shape, and is known as anisocytosis [51]. We also examined megakaryocytes, which give rise to both RBCs and platelets, and observed no differences in platelet frequency in the BM or spleens of *Sost*^−/−^ mice by flow cytometry (Figure 6D). However, we observed an increase in mean platelet volume (MPV) the peripheral blood of in *Sost*^−/−^ mice (Figure 6E). An increase in MPV is usually observed when there is destruction of platelets, commonly seen in myeloproliferative diseases. Interestingly, despite the increase in granulocytes in both the BM and spleen in Scl-Ab-treated mice and WT→*Sost*^−/−^ chimeras, complete blood cell analysis of peripheral blood revealed a significant decrease in neutrophil (the major type of granulocyte) number and frequency (Figure 6E). A comprehensive list of the complete blood count (CBC) data is shown in Table S3.

## 3. Discussion

The sclerostin monoclonal antibody romosozumab is now approved for treatment of osteoporosis in the US, Japan, Canada, Australia, and South Korea) [52,53]. This treatment is effective at treating osteoporosis; however, our previous studies, and our current study in Scl-Ab-treated mice, studies in hematopoietic differentiation in older *Sost*^−/−^ mice, and long-term studies in WT→*Sost^−^*^/−^ chimeras suggest that this treatment may have unintended effects on immune development of patients, many of whom are older and have less plastic immune systems [54]. The safety and efficacy of romosozumab has been assessed, but this assessment has focused mainly on bone mineral density, cardiovascular effects, and risk of hip fractures post-treatment [55]. It is unclear from the clinical trial reports if complete blood counts or similar analysis have been performed; however, our analysis of peripheral blood counts in *Sost*^−/−^ mice did not reveal any effects compared to control mice. Moreover, our original study in 12–15-week-old *Sost*^−/−^ mice [33] did not detect extramedullary hematopoiesis in the spleens. Therefore, it is likely that the effects of sclerostin on hematopoietic cells first occur locally in the BM, and that the functional effects on hematopoietic cells in the context of sclerostin depletion develop relatively slowly, so could be overlooked when monitoring patients treated with romosozumab in the short timeframe analyzed (1 year).

**Figure 7:**
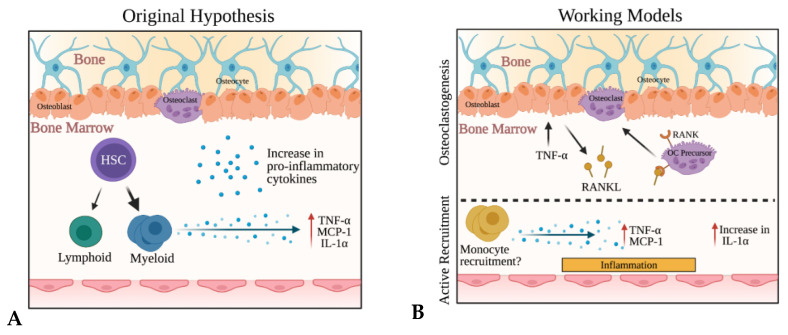
Working models of the mechanisms driving inflammation in the *Sost*^−/−^ bone marrow. (**A**) Inflammation in the *Sost*-depleted bone marrow results in skewed myeloid differentiation at the LT-HSC level, resulting in an increase in monocytes and granulocytes in the bone marrow. The monocytes and granulocytes produce additional inflammatory cytokines. Our results do not support this model. (**B**) Inflammation in the *Sost*^−/−^ bone marrow results in the production of MCP-1 by monocytes in the microenvironment, which, in turn, induces the recruitment of monocytes from the periphery. Monocytes in the bone marrow produce TNFα, further contributing to the inflammation. The source of IL-1α remains unknown. In parallel, the high levels of TNFα in the *Sost*^−/−^ bone marrow may independently promote osteoclastogenesis.

Given that SOST is a known Wnt antagonist and Wnt signaling has been shown to be involved in the regulation of hematopoietic stem cell quiescence and self-renewal [37], we hypothesized that enhanced Wnt signaling in the bone microenvironment would result in an enhancement in HSC self-renewal (proliferation) and numbers. However, our results did not support this hypothesis. Instead, our analyses demonstrated an increase in myeloid cells and evidence of elevated inflammatory cytokines TNFα, MCP-1, and IL-1α in the bone marrow of *Sost*-depleted mice. We posited several possible mechanisms, the first being that *Sost* deficiency influenced a skewing of myeloid differentiation at the HSC level, resulting in an increased production of inflammatory cytokine levels due to increased monocyte and granulocyte numbers (as illustrated in Figure 7, left). Pietras et al. [42] have observed myeloid skewing at the HSC level in experimental models that utilize injection of lipopolysaccharide (LPS) into mice, which induces the production of IFN-γ and IL-1b in the bone marrow. This, in turn, influences differentiation of HSCs into the myeloid lineages at the expense of lymphoid lineages. Since we did not observe any changes in IFN-γ or IL-1b in the bone marrow of *Sost*^−/−^ mice, we reason that the increase in myeloid cell populations in the bone marrow is not due to myeloid-skewed differentiation at the HSC level as described by Pietras et al. Instead, our data are consistent with a negative regulatory role for microenvironmental SOST in the control of TNFα expression and MCP-1 expression in monocytes, which then recruit monocytes and granulocytes to the bone marrow (illustrated in Figure 7, right, bottom). The high levels of MCP-1 may explain the increase in monocytes observed in the *Sost*^−/−^ mice, as MCP-1 and its receptor CCR2 play critical roles in monocyte recruitment during inflammation and bone remodeling [56,57]. It has also been suggested that MCP-1 plays a critical role in the recruitment of monocytes to the bone, as it is induced during osseous inflammation [58]. As the *Sost*^−/−^ microenvironment has been shown to increase myelopoiesis, specifically monocytes, there may be a positive feedback between an increase in monocytes and proinflammatory gene expression. Given that LT-HSCs in WT→*Sost*^−/−^ chimeras upregulate several osteoclast differentiation genes, it is also possible that the high levels of TNFα in the bone marrow induce osteoclast differentiation that is further enhanced in an attempt to restore bone homeostasis in response to the overt osteoblast differentiation occurring in the *Sost*^−/−^ bones (Figure 7, right, top).

The mechanism by which monocytes in the *Sost*^−/−^ mice produce higher levels of TNFα and MCP-1 is still unclear. The relationship between SOST and TNFα is somewhat controversial. In rheumatoid arthritis (RA), an inflammatory disease of the joints in which bone loss is observed, SOST inhibition promotes TNFα-mediated tissue damage, demonstrating a possible protective role of SOST in TNFα-mediated chronic inflammation [59]. The timing of *Sost* upregulation in another RA model suggests that bone loss markers and an increase in *Sost* expression are concomitant with expression of TNFα, preceding arthritis onset [60]. Scl-Ab treatment improves bone loss in RA, further linking SOST and TNFα-mediated inflammation. TNFα induced by inflammation during obesity upregulates *Sost* and contributes to obesity-induced bone loss in mice and in osteocyte cell lines [61]. However, how these studies apply to our results in *Sost*^−/−^ mice, where *Sost* is not expressed globally, is unclear. Since *Sost* is not expressed in *Sost*^−/−^ mice, it cannot initiate the inflammation observed in the bone marrow. In addition, in the RA model [59], *Sost* was produced by synovial tissues, and not colocalized with macrophages or neutrophils. Scl-Ab improves bone loss in RA but does not affect the inflammation in the joints [62]. Therefore, local inflammation may not be an indicator of effects at other sites. Our observation of no differences in TNFα, IL-1α, or MCP-1 in the spleen strongly suggests that the inflammation in *Sost*^−/−^ mice is localized to the bone marrow. In future studies, it may be informative to explore other tissues in the *Sost*^−/−^ mice, as in a rat model of inflammatory bowel disease, which causes systemic inflammation, osteocytes that co-express SOST and TNFα are increased [63].

IL-1α is a dual-function cytokine that can act as a transcription factor, as well as a signal transducer [64]. IL-1α is a classical damage-associated molecular pattern (DAMP) protein produced by damaged or dying cells, and induces inflammation. IL-1α is constitutively expressed in epithelial cells, mesenchymal cell types, and endothelial cells in apoptotic body vesicles. When released into the extracellular space, it can stimulate the attraction of neutrophils [65], and also the attraction of monocytes [66]. Membrane IL-1α is commonly found on the surface of monocytes and B cells, and is critical for IFN-γ activities. Although IL-1α protein levels are clearly elevated in the *Sost^−^*^/−^ mice, we were unable to determine the cellular source of the elevated IL-1α in the current study. Our previous work noted a higher frequency of apoptotic B cells in the bone marrow of *Sost^−^*^/−^ mice [33], and it is possible that these apoptotic B cells express high levels of IL-1α. An increase in *Sost*^−/−^ BM LSKs in early apoptosis was also observed in the current study, which could be another source of IL-1a. ScRNA-seq studies of the bone marrow in wild-type mice show upregulation of IL-1 in VE-cadherin^+^ cells (vessels) and LepR^+^ MSCs (mesenchymal stem cells) after hematopoietic stress induced by 5-fluorouracil [67]. Our scRNA-seq studies focused on residual CD45^+^ cells in collagenase-digested bones, and *IL-1a* was found to be below the level of detection in both control and *Sost^−^*^/−^ mice, in both CD45^−^ and CD45^+^ cells. The absence of *IL-1a* expression was surprising, since it is expressed by a wide variety of cells. At this time, we are unable to conclude if this is a technical issue specific to *IL-1a* detection using scRNA-seq. Therefore, it is possible that *IL-1a* in the *Sost^−^*^/−^ mice is produced by both nonhematopoietic stromal cells, as well as hematopoietic cells, but further studies are necessary to resolve this in detail. It would be interesting to utilize IL-1 antagonists in vivo in *Sost^−^*^/−^ mice and Scl-Ab-treated animals to explore if the bone marrow inflammation is reduced or prevented.

We note that the inflammatory cytokine changes were not observed in younger *Sost*^−/−^ mice (data not shown). Therefore, it is also possible that Sost deficiency may also contribute to “inflammaging”, defined as chronic, sterile, low-grade inflammation that contributes to the pathogenesis of age-related diseases [68]. “Inflammaging” is characterized by immunosenescense and thrombocytosis [23], as well as overproduction of systemic inflammatory cytokines IL-1, TNFα, IL-6, and C-reactive protein (CRP) [24]. One of the driving forces of inflammaging is believed to be chronic stimulation of the immune system. It is now widely accepted that myeloid-biased differentiation of HSCs is associated with aging and chronic inflammation [69]. Increased inflammation during aging can result in concurrent decline in HSC function. The decline of HSC functionality includes a reduction in long-term repopulating potential, homing and engraftment after transplant [19], a decrease in lymphopoiesis, and increase in myelopoiesis [20,21], as well as an increase in the total number of LT-HSCs [22]. Chronic inflammation has also been associated with aging, characterized by anemia, immunosenescense, and thrombocytosis [23], as well as overproduction of inflammatory cytokines IL-1, TNFα, and IL-6 [24]. Our *Sost*^−/−^ mice are particularly interesting, as they have chronic low-grade local inflammation, but no changes in immunosenescense or thrombocytosis.

Another feature of inflammaging is in the induction of EMH in the spleen, where we also observed an increase in myelopoiesis. The observation of increased frequency of proliferative, live LSKs and decrease in LSKs in late apoptosis stages in the spleen is consistent with the EMH observed in the *Sost*^−/−^ mice. Paradoxically, the splenic LSKs also displayed an increase in early apoptosis. It is possible that the rates of proliferation exceed the rates of apoptosis in the LSKs in the *Sost*^−/−^ mice, resulting in a net increase in LSKs. Our current analyses only reflect a snapshot of the proliferation and apoptosis status of the cell at a single timepoint. Further investigation is required to reconcile this, perhaps by direct measurements of LSK output using BrdU labeling long-term and analysis of apoptosis as a function of time over the lifespan of the *Sost*^−/−^ mice.

It is known that inflammation induces adipogenesis [70]. It has also been shown that *Sost*^−/−^ mice display reduced BM adipose tissue (BMAT) at 6 weeks of age [71]. As we do not see an increase in inflammation in young *Sost*^−/−^ mice, it would be interesting to assess BMAT at later ages in *Sost*^−/−^ mice. If the trend of decreased BMAT in *Sost*^−/−^ mice stays the same, this would directly contradict the known mechanisms of inflammation and adipogenesis. *Sost*^−/−^ mice also display a decrease in B cell development; however, decreased B cell development is correlated with an increase in adiposity in the bone marrow [72,73]. Osteocytes and bone marrow adipocytes play direct roles in the maintenance of B cell development, and SOST is an important regulator of these cellular interactions in the bone. Since MSCs are a common progenitor of both adipocytes and osteocytes, it is also possible that SOST acts directly at the level of the MSC [74]. It is tempting to speculate that deletion of *Sost* could affect MSC maintenance and function, which, in turn, could affect HSCs and B cells within the bone marrow niches [75], independently of the status of the BM adiposity and bone mass. Further work is necessary to ascertain the effects of constitutive marrow adipose tissue (cMAT) and regulated marrow adipose tissue (rMAT) on cellular metabolism during hematopoiesis [76], and if cMAT and rMAT are regulated by Sost.

Inflammation in the bone marrow has been shown to have many effects on erythrocyte development. The significant decrease in mature splenic RBCs in *Sost*^−/−^ mice may represent a direct consequence of inflammaging. Prior studies have shown that TNFα inhibits erythropoiesis directly through activation of GATA-2, whose overexpression is known to inhibit erythropoiesis in favor of megakaryopoiesis [77,78]. We do not, however, observe an increase in platelet production in *Sost*^−/−^ mice. The most common effect of inflammation on erythropoiesis is anemia, as defined by decreased hemoglobin levels. This is notable as *Sost*^−/−^ mice do not have any discernable differences in hemoglobin levels in the blood. During inflammation, it is known that RBCs adhere to the endothelium due to increased expression of endothelial adhesion molecules [79]. Additionally, during periods of increased erythrocyte destruction, erythrophagocytosis and iron recycling are primarily carried out by hepatic macrophages differentiating in the liver from circulating monocytes, rather than by resident splenic macrophages [80]. It may be of interest to investigate the specific monocyte populations present in the bone marrow and liver in future studies.

Our analysis of erythrocyte development in the peripheral blood of *Sost^−^*^/−^ mice revealed a significant increase in RDW, known as anisocytosis [81]. RDW is elevated in folate deficiencies characteristic of macrocytic anemia [82]. Although folate levels in *Sost*^−/−^ mice remain untested, decreased folate levels have been associated with poor bone health in humans [83]. Anisocytosis can be split into two groups: anisocytosis with microcytosis, characterized by low iron (e.g., sickle cell anemia), and anisocytosis with macrocytosis, characterized by folate deficiency (e.g., myelodysplastic syndrome). We know that *Sost*^−/−^ mice have normal hemoglobin levels (Table S2), indicating no issues with iron; however, iron levels in *Sost*^−/−^ mice have not been directly quantified to date. The increase in mean platelet volume (MPV) in the blood is usually associated with an increase in platelet production, or destruction of platelets, as seen in myeloproliferative diseases [84,85,86], such as chronic myeloid leukemia (CML), myelofibrosis, and myelodysplastic syndrome (MDS) [87,88,89]. *Sost*^−/−^ mice displayed no changes in the blood platelet levels (Table S2). Future studies are needed to investigate if increased MPV is related to the increased myelopoiesis observed in *Sost*^−/−^ mice.

Reduced capacity for erythropoiesis or platelet production is often compensated by extramedullary hematopoiesis in the spleen [90]. The induction of EMH in the spleens of *Sost*^−/−^ mice with age seems to suggest an attempt at compensation for the decrease in RBC production in the spleen. As *Sost* is not expressed in the spleen, the physiological mechanisms that drive extramedullary hematopoiesis in the spleens of the *Sost*^−/−^ mice is of particular interest to our laboratory. The decreased expression of CXCL12 in the BM of *Sost*^−/−^ mice suggests that LT-HSCs may be unable to be retained in the BM and are actively migrating to the spleen. Certainly, the biology of splenic hematopoietic niches is not well understood, and future studies could determine if LT-HSCs in *Sost*^−/−^ mice are actively migrating from the BM and into the spleen or if LT-HSCs are expanding in the spleen de novo. Future studies to understand, identify, and test the function of stromal cells in the spleen that drive extramedullary hematopoiesis in *Sost*^−/−^ mice.

Our study revealed important similarities and differences between the hematopoietic development in the acute Sost depletion (Scl-Ab) model, the chronic *Sost* depletion (LT-HSC transplantation) model, and the global *Sost* knockout mouse model [33]. Granulocytes were markedly increased in all three models, but monocyte frequency was increased only in the chronically depleted, WT→*Sost*^−/−^chimeras. One possible explanation for this is that the kinetics of monocyte responses may take longer to emerge than the time we observed in the acute Scl-Ab model of sclerostin depletion. To reconcile these differences, future studies could include the use of an inducible *Sost*^−/−^ model to knock out *Sost* in adult mice for a specified amount of time, instead of from ontogeny. This would allow similar timing for sclerostin depletion in both the genetic knockout and Scl-Ab treatment. Additional studies are also required to examine if the changes in hematopoiesis are reversed after Scl-Ab treatment is terminated, similar to the loss of bone mass reported post-Scl-Ab treatment, observed in mice and monkeys [91,92].

## 4. Materials and Methods

Study Design/Statistical Analysis. G*Power 3.1 software [93] was used to calculate the sample size required per group for all experiments. Specifically, the type of power analysis used was a priori: compute required sample size given α, power, and effect size, where n_1_ = n_2_ (allocation ratio N_2_/N_1_ = 1), α = 0.05, with two tails, normal parent distribution, and effect size of d = 0.5. All box and whisker plots are composed with the ends of the box corresponding to the upper and lower quartiles, with the median marked by the vertical line inside. The whiskers correspond to the highest and lowest observations. All line data were expressed as the mean ± standard deviation. For flow cytometry results, statistical analysis was done using Mann–Whitney U-test or Student’s *t*-test with a two-tailed distribution, with two-sample equal variance (homoscedastic test) using GraphPad Prism 9 (San Diego, CA, USA). For all tests, *p* < 0.05 was considered to be statistically significant.

Mice. C57BL/6 mice (Stock No: 000664) and B6.SJL (CD45.1, Stock No: 002014) were purchased from The Jackson Laboratory(Bar Harbor, ME). *Sost*^−/−^ LacZ knock in mice, in which *Sost* is globally deleted, have been previously described [33,74]. Mice of both sexes were used on the C57BL/6 background and housed in sterile, microisolator cages with autoclaved feed and water. The UC Merced or Lawrence Livermore National Laboratories IACUCs approved all animal work (Protocol #19-0004, approved 7 March 2019).

Flow cytometric analysis of bone marrow and splenic cells. Procedures for euthanasia, dissection, and preparation of femurs and tibias were performed as previously described [33]. Antibodies used are listed in Table S4 and were purchased from eBioscience (San Diego, CA, USA), BioLegend (San Diego, CA, USA), Miltenyi Biotec (Auburn, CA, USA), and BD Biosciences (San Jose, CA, USA). A list of cell surface markers for the cell populations we examined is provided in Table S5. Staining of all cells included a preincubation step with unconjugated anti-CD16/32 (clone 2.4G2 or clone 93) mAb to prevent nonspecific binding of mAbs to Fc*γ*R, except when staining for CD16/32. For extracellular staining, the cells were incubated with a panel of biotinylated mAbs for 20 min on ice, followed by a secondary stain with fluorochrome-conjugated streptavidin and additional primary directly conjugated mAbs for 20 min on ice, and then 10 min at room temperature protected from light. DAPI or PI was used as a viability dye. Single color stains were used for setting compensations and gates were determined by historical data, in addition to fluorescence-minus-one control stains. Flow cytometric data were acquired on the BD LSR II flow analyzer or BD Aria III flow sorter, depending on the experimental procedure. The raw flow cytometry data were analyzed using Flowjo, version 10 (FlowJo LLC, Ashland, OR, USA).

Annexin V staining. Five million cells per sample were stained with anti-Lineage, Sca-1, and cKit antibodies for 20 min on ice and 10 min at RT in the dark. Cells were then washed and stained with annexin V 1:50 in annexin binding buffer and incubated for 30 min at RT in the dark. Cells were washed with annexin binding buffer and PI was added before acquisition.

Ki67 staining. Cells were extracellularly stained with anti-Lineage, Sca-1, and cKit antibodies, and then fixed and permeabilized using the eBioscience™ Foxp3/Transcription Factor Staining Buffer Set for intranuclear staining. Cells were stained on ice for 30 min in the dark with anti-Ki67-PE (clone SolA15) or isotype-PE control mAb, purchased from eBioscience. After incubation, cells were washed twice with permeabilization buffer, resuspended in 200 µL FACS buffer, and transferred to tubes for acquisition on the LSR II. DAPI was added to the samples at a final concentration of 1.0 ug/mL immediately before acquisition.

Scl-Ab treatment. SOST antibody (Scl-Ab; a gift from Regeneron) (25 mg/kg) or vehicle (PBS) was administered subcutaneously to C57BL/6 mice, starting at 8 weeks of age, twice weekly for 6 weeks.

uCT analysis. Formalin-fixed femora from vehicle- and Scl-Ab-treated mice were scanned, reconstructed, and analyzed as previously described [94]. Briefly, 10-μm resolution, 50-kV peak tube potential and 151-ms integration time were used to collect scans on a Scanco uCT-35 tomographer. The distal 60% of each femur was scanned. Standard morphometric and structural parameters related to cancellous and cortical bone architecture were measured [95].

Transplantation. To purify LT-HSCs for transplant, enrichment of LT-HSCs from WT B6.SJL mice was performed using a biotinylated anti-“lineage” antibody cocktail (anti-CD3, CD4, CD8, CD11b, CD19, Gr1, NK1.1, and Ter119), followed by magnetic separation using EasySep Positive Selection kit (Stem Cell Technologies, Vancouver BC). After enrichment, cells were stained with streptavidin Pacific Blue and antibodies to Sca1, cKit, CD150, and CD48. Cells were aseptically sorted on the BD FACS Aria II cell sorter to a 90.3–100% purity. Equivalent numbers of LT-HSCs (minimum of 225) were injected retro-orbitally into sub-lethally irradiated (750 rads with a ^137^Cs source, J.L. Shepherd and Associates, San Fernando, CA, USA) control B6 or *Sost*^−/−^ recipients under isoflurane anesthesia. Animals were supplemented with neomycin in the drinking water for 14 days post-transplant and analyzed for chimerism in the peripheral blood every 6 weeks, as described [33]. Recipient mice were 16–22 weeks of age at the time of transplantation, and their bone marrow was analyzed at 16–20 weeks post-transplantation.

Quantification of cytokines. Long bones were dissected and cleaned of muscle, and epiphyses removed. The bones were then placed in a 0.5 mL tube, in which a hole was introduced in the bottom using an 18 g needle. One marrow serum was harvested by removing epiphyses from both long bones after dissection and removal of muscle, placing bones in a 0.5 mL tube with 60 µL PBS, with a hole in the bottom from an 18 gauge needle. To harvest the BM serum, the 0.5 mL tube containing the bone was then placed into a 1.5 mL microcentrifuge tube, and 60 µL PBS was added to the bone. Tubes were centrifuged together for 30 s at 10,000× *g*, collecting the serum supernatant in the 1.5 mL tube. Spleen serum was harvested by placing the spleen into a 1.5 mL tube with 60 µL PBS and homogenizing for 1 min. The sample was centrifuged for 30 s at 10,000× *g*, and the serum supernatant collected. For peripheral blood collection, mice were heated gently under a heat lamp and 7–8 drops of blood were collected from the tail veins in nonheparinized tubes. Blood was allowed to clot for at least 30 min, and then was centrifuged for 10 min at 1000× *g* to separate the serum from the blood cells. Concentrations of cytokines (IL-1α, IL-1β, IL-6, IL-10, IL-12p70, IL-17A, IL-23, IL-27, MCP-1, IFN-β, IFN-γ, TNFα, and GM-CSF) were determined from the serum of bone marrow, spleen, and blood using the LEGENDplex™ Mouse Inflammation Panel from Biolegend, Inc. according to the manufacturer’s instructions.

Bulk RNA-seq and data analysis. Donor-derived LT-HSCs pooled from WT→ and WT→*Sost*^−/−^ bone marrow chimeras were sorted using flow cytometry for bulk RNA-sequencing analysis. Cells were sorted into Buffer RLT lysis buffer (Qiagen, Redwood City, CA, USA) supplemented with β-mercaptoethanol, followed by RNA isolation using RNeasy Micro kit (Qiagen) per the manufacturer’s protocol. Sequencing library preparation was performed using SMART-Seq v4 Ultra Low Input RNA Kit (Takara Bio, San Jose, CA, USA) according to the manufacturer’s protocol, then sequenced on an Illumina NextSeq 500. Sequence reads were aligned to mouse genome (mm10) using TopHat2 [96], and gene-wise read counts were generated using “featureCounts” [97]. Subsequently, data were normalized using “TMM” normalization method, and differentially expressed genes were identified with “limma” using “voom” method [98]. Enriched “biological processes” associated with differentially expressed genes were identified using ToppGene [99]. Heatmaps and dot plots were generated using custom R scripts.

Single-cell RNA-seq and data analysis. Single-cell preparation of bone-derived cells were obtained from freshly dissected long bones (femurs and tibias) from 10-week-old mice per genotype (n = 2). Following a thorough removal of superficial tissue, bones were cut at the epiphysis and bone marrow was flushed with 10 mL wash buffer (DMEM/F12 + 1%BSA) using a 29 gauge needle inserted into the marrow. Flushed bones were gently crushed with a pestle in 5 mL of fresh wash buffer to yield 1–2 mm in diameter bone fragments, followed by an additional 5 mL wash in wash buffer. Bone fragments were then digested in 5 mL of 3 mg/mL collagenase type I (Worthington Biochemicals, Lakewood, NJ, USA) and 100 µg/mL DNase I (Roche, Basel, Switzerland) in wash buffer for 45 min at 37 °C with shaking (120 rpm). All supernatants were collected and stored on ice with supplementation of equal volume complete (DMEM/F12 with 10% FBS) until final processing. Next, 5 mL of 0.25% trypsin–EDTA (ThermoFisher Scientific, Agawam, MA, USA) were added to bone fragments for 10 min at 37 °C with shaking (120 rpm) before collection of supernatant, followed by a wash and collection with 5 mL of wash buffer. Pooled supernatants/wash were centrifuged at 500× *g* for 10 min at 4 °C, then resuspended in complete media on ice. Collagenase/trypsin digestions were performed 2 additional times with all cells pooled, followed by a red blood cell removal using ACK lysing buffer (ThermoFisher Scientific), then final resuspension in PBS with 0.04% nonacetylated BSA. Single-cell library preparation was performed using Chromium Single Cell 3′ GEM, Library & Gel Bead Kit v3 (10× Genomics, catalog no. 1000075, Pleasanton, CA, USA) following the manufacturer’s protocol. Libraries were sequenced on Illumina NextSeq500. We then used 10× Genomics Cell Ranger pipeline for sample demultiplexing, barcode processing, aligning to the mouse genome (mm10), and quantifying gene expression. Further analysis was performed in R using the Seurat package [100]. First, cells with fewer than 500 detected genes per cell and genes that were expressed by fewer than 5 cells were filtered out. Subsequently, the data were normalized using a global-scaling normalization method “LogNormalize”. Then, WT and *Sost*^−/−^ datasets were anchored and integrated using the top 2000 variable features per dataset calculated via the “vst” method, and a new integrated matrix was generated. The integrated data were then scaled to a mean of 0 and variance of 1, and the dimensionality of the data was reduced by principal component analysis (PCA, GA, USA). The first principal components were then used for dimensionality reduction using uniform manifold approximation and projection (UMAP), and clustering using the Louvain algorithm. To further characterize immune cells, we extracted all cells with robust *Ptprc* (CD45) expression and analyzed as described above. This analysis resulted in 10 immune clusters. Cluster markers were identified using the “FindAllMarkers” function in Seurat.

Hematoxylin and eosin (H&E). Spleen samples were placed in 10% buffered formalin for 24 h. Samples were then dehydrated with increasing concentrations of ethanol, cleared with xylene, then placed in wax and embedded in a mold. Samples were then sliced via microtomy, placed on slides, and deparaffinized, before staining with hematoxylin followed by eosin.

Complete blood counts. Peripheral blood was collected either by femoral vein blood collection immediately following cervical dislocation, or tail vein blood collection following heating under a heat lamp. In both cases, blood was collected in heparinized tubes. Complete blood counts were evaluated within 6 h after collection on a Hemavet 950 Veterinary Hematology System.

## 5. Conclusions

In summary, our studies extend previous studies and reveal novel information on the effects of sclerostin deficiency in bone and sclerostin-depleting treatments on hematopoietic stem cells. Our data indicate that SOST depletion and *Sost* deficiency not only affects B cell development in the BM relatively early, but also creates an inflammatory bone marrow microenvironment that may become more severe over time. Investigation of the changes in the bone marrow architecture, including alterations in vasculature function and quality, proportions of arterioles and sinusoidal vessels, and local oxygen tension [67,101,102,103], may provide evidence for the specific cell-extrinsic mechanisms that drive the promotion of myelopoiesis in the *Sost*^−/−^ mice and Scl-Ab-treated mice. In future studies, it would be interesting to investigate the localization of HSC in the *Sost*^−/−^ bone marrow endosteal and sinusoidal niches, and quantify if specialized niches that promote myeloid differentiation or recruitment are increased [104]. Studies to test possible combinational therapy of Scl-Ab with IL-1 antagonists or TNFα blockade to control myeloid skewing are another potential area of further investigation. The information provided by our studies may be useful in monitoring humans treated with romosozumab for changes in immune cell frequencies, chronic inflammation, and signs of anemia, such that treatments for osteoporosis can be modified to address these hematopoietic changes.

## Supplementary Materials

The following are available online at https://www.mdpi.com/article/10.3390/ijms22179111/s1, Figure S1. Sclerostin-depleting antibodies change hematopoietic differentiation; Figure S2. Analysis of LT-HSC frequency and cellularity in *Sost*^−/−^ mice; Figure S3. Proliferation and cell death are altered in *Sost^−/^*^−^ bone marrow and spleens; Figure S4. Hematopoietic progenitors derived from *Sost*^−/−^ bone marrow display normal hematopoiesis after transplantation into a WT microenvironment. Figure S5. RNA-Seq heatmap results; Figure S6. ScRNA-Seq Analysis of immune cells from the long bones from WT and *Sost*^−/−^ mice; Figure S7. Analysis of CXCL12 and SCF in bone stromal cells of *Sost*^−/−^ mice; Figure S8. Evidence of extramedullary hematopoiesis in *Sost*^−/−^ mice; Figure S9. LT-HSCs from the spleen show no functional differences compared to the bone marrow; Table S1. Ontologies associated with genes highly expressed in LT-HSCs sorted from WT→*Sost*^−/−^ hematopoietic chimeras compared to LT-HSCs sorted from WT→WT hematopoietic chimeras; Table S2. Gene lists for selected upregulated ontologies highly expressed in LT-HSCs sorted from WT→*Sost*^−/−^ hematopoietic chimeras compared to LT-HSCs sorted from WT→WT hematopoietic chimeras; Table S3. Complete blood count values; Table S4. Antibodies used for flow cytometry; Table S5. List of Cell Surface Markers used for Flow Cytometry.
