# Supplementary material for: Sclerostin Depletion Induces Inflammation in the Bone Marrow of Mice
Source: Int J Mol Sci. 2021 Aug 24;22(17):9111. doi: 10.3390/ijms22179111 (PMC8431516; doi:10.3390/ijms22179111)
Supplement: Supplementary file 1 [file ijms-22-09111-s001.zip › ijms-1346622-supplementary.pdf]

SUPPLEMENTARY MATERIALS

Supplemental Materials and Methods

Bone digestion for quantitative real-time PCR. Bone digests to obtain MSC, OBs, ECs and enriched osteocytes was performed similar to Schepers et al. (1). Femurs and tibias were harvested in 1x HBSS without serum and crushed using a mortar and pestle. Bone marrow was washed away and the bone chips were then incubated with 3mg/ml Type I Collagenase in HBSS without serum (Worthington) at 37°C on a shaker at 110 rpm for 1 hour. After incubation the supernatant was harvested from the bone chips, filtered through a 70 um cell strainer, and then washed with 1x HBSS-2% FCS to stop the action of the collagenase. Cells were centrifuged at 300-500 x g for 5 minutes to wash, resuspended in 1x HBSS-2% FCS, and cell counts performed using Trypan Blue staining on a hematocytometer. Cells were then stained for flow cytometry with antibodies to lineage-positive cells (CD3, CD4, CD8, CD11b, CD19, NK1.1, GR1 and TER119, all in PE-Cy7), CD45-eFluor450, Sca1-FITC, CD31-APC, CD51-biotin followed by streptavidin-PE. PI was added as viability marker. Cells were sorted using a FACS Aria III and cell pellets stored for real-time PCR.

Real-time PCR. LT-HSCs were purified from pooled BM from control and *Sost*$^{-/-}$ long bones by flow cytometry, as described in the main text. Cells were pelleted and resuspended in RNeasy Lysis Buffer with 2-mercaptoethanol (Qiagen). Total RNA was purified using Qiagen RNeasy Micro Kit (Qiagen) according to manufacturer's protocol. RNA concentration and purity was analyzed using a NanoDrop ND-1000 Spectrophotometer (Thermo Fisher Scientific). The same amount of RNA from each sample was mixed with qScript XLT-1 Step, RT-qPCR ToughMix (Quantabio Cat. 66149433) together with specific TaqMan expression assays. Real-time quantitative PCR was ran on an Applied Biosystems thermocycler in this sequence: 1 cycles at 50°C for 10 minutes for cDNA synthesis, 1 cycle at 95°C for 1 minute for initial denaturation and then 40 cycles of amplification at 95°C for 5 seconds then 60°C for 45 seconds, using QuantStudio 3 software (ThermoFisher). The following TaqMan gene expression assays (Thermo Fisher Scientific) were used: HoxB4-FAM (Mm00657964_m1), p21/Cip1/Cdkn1a-FAM (Mm00432448_m1), and GAPDH-VIC (MM999915_g1). Expression of HoxB4 and p21/Cip1 in KO LT-HSCs relative to the average WT gene expression was calculated using the double delta Ct method (2). RNA from digested bones and qPCR for CXCL12 and SCF was performed as described in Cain and Manilay (3).

Cell cycle analysis after 5-fluorouracil treatment. B6 and *Sost*$^{-/-}$ mice (16-18 weeks of age) were injected with PBS or 100mg/kg 5-fluorouracil (Sigma) intraperitoneally. Seven days later, mice were euthanized and bone marrow cells were harvested for flow cytometry as described above. First, cells were

extracellularly stained with anti-Lineage, Sca-1 and cKit antibodies, and then fixed and permeabilized using the eBioscience™ Foxp3 / Transcription Factor Staining Buffer Set for intranuclear staining. Cells were stained on ice for 30 minutes in the dark with anti-Ki67-PE (clone SolA15) or isotype-PE control mAb, purchased from eBioscience. After incubation, cells were washed twice with permeabilization buffer, resuspended in 400µl FACS buffer and transferred to tubes for acquisition on the LSR II. DAPI was added to the samples at a final concentration of 1.0 ug/ml immediately before acquisition.

Table S1. Ontologies associated with genes highly expressed in LT-HSCs sorted from WT→*Sost*$^{-/-}$ hematopoietic chimeras compared to LT-HSCs sorted from WT→WT hematopoietic chimeras

GO ID	Term	FDR	No of genes
GO:0002526	acute inflammatory response	2.24E-07	17
GO:0002250	adaptive immune response	1.19E-12	56
GO:0002460	adaptive immune response based on somatic recombination of immune receptors built from immunoglobulin superfamily domains	5.15E-11	38
GO:0046631	alpha-beta T cell activation	2.16E-09	23
GO:0001525	angiogenesis	1.27E-08	44
GO:0042113	B cell activation	2.41E-15	41
GO:0042100	B cell proliferation	1.60E-09	18
GO:0050853	B cell receptor signaling pathway	5.20E-07	17
GO:0022610	biological adhesion	2.13E-18	99
GO:0001568	blood vessel development	9.60E-08	50
GO:0048514	blood vessel morphogenesis	6.13E-09	49
GO:0045453	bone resorption	1.30E-06	13
GO:0035710	CD4-positive, alpha-beta T cell activation	1.09E-07	17
GO:0001775	cell activation	1.38E-51	153
GO:0002263	cell activation involved in immune response	3.99E-30	83
GO:0007155	cell adhesion	1.58E-18	99
GO:0060326	cell chemotaxis	7.63E-10	32
GO:0016477	cell migration	7.06E-12	93
GO:0098609	cell-cell adhesion	1.18E-13	66
GO:0006968	cellular defense response	2.25E-06	11
GO:0019725	cellular homeostasis	1.74E-07	58
GO:0006873	cellular ion homeostasis	2.17E-06	43
GO:0062197	cellular response to chemical stress	1.31E-06	29
GO:0071345	cellular response to cytokine stimulus	1.02E-17	83
GO:0034599	cellular response to oxidative stress	1.01E-06	27
GO:1901701	cellular response to oxygen-containing compound	5.73E-07	64
GO:0048878	chemical homeostasis	3.27E-07	66
GO:0006935	chemotaxis	1.32E-10	50
GO:0001816	cytokine production	5.73E-17	73
GO:0019221	cytokine-mediated signaling pathway	7.12E-12	57
GO:0006952	defense response	1.32E-26	128
GO:0098542	defense response to other organism	2.93E-10	71
GO:0140029	exocytic process	1.95E-21	80
GO:0006887	exocytosis	1.95E-21	80
GO:0140352	export from cell	1.17E-22	107
GO:0036230	granulocyte activation	6.75E-27	65
GO:0071621	granulocyte chemotaxis	3.28E-08	19

GO:0097530	granulocyte migration	2.41E-08	21
GO:0048534	hematopoietic or lymphoid organ development	3.86E-21	86
GO:0030097	hemopoiesis	1.57E-20	83
GO:0006959	humoral immune response	3.05E-08	33
GO:0002252	immune effector process	7.47E-33	117
GO:0002520	immune system development	9.98E-21	88
GO:0006954	inflammatory response	6.12E-24	79
GO:0045087	innate immune response	8.63E-09	59
GO:0098771	inorganic ion homeostasis	2.43E-06	46
GO:0050801	ion homeostasis	1.68E-06	48
GO:0045321	leukocyte activation	4.78E-53	147
GO:0002366	leukocyte activation involved in immune response	3.11E-30	83
GO:0071887	leukocyte apoptotic process	2.43E-06	16
GO:0007159	leukocyte cell-cell adhesion	6.37E-18	47
GO:0030595	leukocyte chemotaxis	4.13E-10	28
GO:0043299	leukocyte degranulation	1.03E-26	67
GO:0002521	leukocyte differentiation	2.62E-22	65
GO:0002443	leukocyte mediated immunity	6.05E-32	95
GO:0050900	leukocyte migration	5.88E-16	52
GO:0070661	leukocyte proliferation	8.63E-19	46
GO:0046649	lymphocyte activation	1.44E-28	86
GO:0002285	lymphocyte activation involved in immune response	9.81E-07	21
GO:0030098	lymphocyte differentiation	4.93E-14	43
GO:0002449	lymphocyte mediated immunity	7.17E-08	32
GO:0046651	lymphocyte proliferation	3.55E-17	42
GO:0000165	MAPK cascade	2.51E-08	57
GO:0061025	membrane fusion	1.28E-19	84
GO:1903131	mononuclear cell differentiation	3.19E-16	49
GO:0071674	mononuclear cell migration	1.82E-06	20
GO:0032943	mononuclear cell proliferation	1.40E-18	44
GO:0048871	multicellular organismal homeostasis	4.44E-07	38
GO:0072674	multinuclear osteoclast differentiation	9.73E-07	6
GO:0002275	myeloid cell activation involved in immune response	5.00E-26	67
GO:0030099	myeloid cell differentiation	9.85E-11	41
GO:0002274	myeloid leukocyte activation	2.11E-32	83
GO:0002573	myeloid leukocyte differentiation	1.36E-12	31
GO:0002444	myeloid leukocyte mediated immunity	1.25E-28	71
GO:0097529	myeloid leukocyte migration	6.83E-09	26
GO:0050866	negative regulation of cell activation	5.66E-08	24
GO:0007162	negative regulation of cell adhesion	1.39E-08	30
GO:0022408	negative regulation of cell-cell adhesion	1.22E-06	21

GO:0001818	negative regulation of cytokine production	6.74E-07	30
GO:0002683	negative regulation of immune system process	4.72E-13	43
GO:0002695	negative regulation of leukocyte activation	6.62E-09	24
GO:0051250	negative regulation of lymphocyte activation	3.55E-08	21
GO:0051241	negative regulation of multicellular organismal process	1.11E-06	62
GO:0032102	negative regulation of response to external stimulus	1.28E-08	35
GO:0048585	negative regulation of response to stimulus	3.88E-07	84
GO:0042119	neutrophil activation	3.64E-27	65
GO:0002283	neutrophil activation involved in immune response	1.64E-25	62
GO:0030593	neutrophil chemotaxis	5.88E-08	17
GO:0043312	neutrophil degranulation	1.03E-25	62
GO:0002446	neutrophil mediated immunity	7.59E-28	66
GO:1990266	neutrophil migration	3.28E-08	19
GO:0048284	organelle fusion	1.38E-19	81
GO:0090174	organelle membrane fusion	7.76E-20	80
GO:0030316	osteoclast differentiation	2.19E-06	15
GO:0006909	phagocytosis	4.55E-08	32
GO:0006911	phagocytosis, engulfment	1.85E-06	16
GO:0002675	positive regulation of acute inflammatory response	1.25E-07	10
GO:0043085	positive regulation of catalytic activity	4.58E-07	73
GO:0050867	positive regulation of cell activation	5.80E-14	45
GO:0045785	positive regulation of cell adhesion	2.68E-13	45
GO:0010647	positive regulation of cell communication	4.16E-10	93
GO:0008284	positive regulation of cell population proliferation	9.15E-08	59
GO:0022409	positive regulation of cell-cell adhesion	2.88E-14	37
GO:0001819	positive regulation of cytokine production	6.95E-10	41
GO:0031349	positive regulation of defense response	1.22E-07	31
GO:0051094	positive regulation of developmental process	3.43E-07	71
GO:0010628	positive regulation of gene expression	1.20E-08	68
GO:1903708	positive regulation of hemopoiesis	1.95E-11	26
GO:0002922	positive regulation of humoral immune response	1.31E-06	8
GO:0002699	positive regulation of immune effector process	2.58E-07	24
GO:0050778	positive regulation of immune response	1.32E-10	54
GO:0002684	positive regulation of immune system process	6.11E-21	87
GO:0050729	positive regulation of inflammatory response	7.98E-10	22
GO:1902533	positive regulation of intracellular signal transduction	5.67E-11	67
GO:0002696	positive regulation of leukocyte activation	1.58E-14	45
GO:1903039	positive regulation of leukocyte cell-cell adhesion	2.11E-13	33
GO:1902107	positive regulation of leukocyte differentiation	1.95E-11	26
GO:0070665	positive regulation of leukocyte proliferation	2.28E-06	18
GO:0051251	positive regulation of lymphocyte activation	7.57E-12	38

GO:0045621	positive regulation of lymphocyte differentiation	1.02E-06	16
GO:0043410	positive regulation of MAPK cascade	2.58E-07	39
GO:0051240	positive regulation of multicellular organismal process	3.57E-13	92
GO:0045639	positive regulation of myeloid cell differentiation	6.69E-08	17
GO:0002763	positive regulation of myeloid leukocyte differentiation	1.91E-06	12
GO:0045937	positive regulation of phosphate metabolic process	5.75E-07	57
GO:0010562	positive regulation of phosphorus metabolic process	5.75E-07	57
GO:0042327	positive regulation of phosphorylation	2.43E-07	55
GO:0001934	positive regulation of protein phosphorylation	8.88E-08	53
GO:0032103	positive regulation of response to external stimulus	8.86E-09	40
GO:0009967	positive regulation of signal transduction	8.86E-10	85
GO:0023056	positive regulation of signaling	4.61E-10	93
GO:0050870	positive regulation of T cell activation	1.32E-10	28
GO:0051050	positive regulation of transport	2.19E-06	54
GO:0006468	protein phosphorylation	1.35E-06	77
GO:0045055	regulated exocytosis	7.67E-22	75
GO:0002673	regulation of acute inflammatory response	1.74E-07	12
GO:0002819	regulation of adaptive immune response	3.33E-07	21
GO:0046634	regulation of alpha-beta T cell activation	5.92E-07	16
GO:0045765	regulation of angiogenesis	2.39E-06	28
GO:0050864	regulation of B cell activation	2.57E-10	26
GO:0050865	regulation of cell activation	1.17E-25	75
GO:0030155	regulation of cell adhesion	3.11E-19	70
GO:0045595	regulation of cell differentiation	5.53E-08	84
GO:0042127	regulation of cell population proliferation	1.45E-12	100
GO:0022407	regulation of cell-cell adhesion	9.93E-17	50
GO:0001817	regulation of cytokine production	5.80E-14	64
GO:0031347	regulation of defense response	4.65E-13	57
GO:0002604	regulation of dendritic cell antigen processing and presentation	1.90E-06	6
GO:0071622	regulation of granulocyte chemotaxis	3.14E-07	12
GO:1903706	regulation of hemopoiesis	2.76E-14	46
GO:0002697	regulation of immune effector process	1.65E-10	42
GO:0050776	regulation of immune response	5.45E-19	80
GO:0002682	regulation of immune system process	7.47E-33	132
GO:0050727	regulation of inflammatory response	1.02E-13	42
GO:0002694	regulation of leukocyte activation	1.65E-27	75
GO:2000106	regulation of leukocyte apoptotic process	8.20E-07	15
GO:1903037	regulation of leukocyte cell-cell adhesion	1.34E-16	43
GO:0002688	regulation of leukocyte chemotaxis	3.80E-07	17
GO:1902105	regulation of leukocyte differentiation	6.85E-14	38
GO:0002685	regulation of leukocyte migration	1.30E-08	25

GO:0070663	regulation of leukocyte proliferation	6.48E-13	33
GO:0051249	regulation of lymphocyte activation	3.05E-22	62
GO:0045619	regulation of lymphocyte differentiation	1.45E-09	25
GO:0050670	regulation of lymphocyte proliferation	2.07E-12	31
GO:0043408	regulation of MAPK cascade	1.66E-06	45
GO:0032944	regulation of mononuclear cell proliferation	4.72E-13	32
GO:2000026	regulation of multicellular organismal development	1.70E-11	86
GO:0045637	regulation of myeloid cell differentiation	4.47E-08	27
GO:0002761	regulation of myeloid leukocyte differentiation	4.08E-08	19
GO:0090022	regulation of neutrophil chemotaxis	2.97E-07	10
GO:1902622	regulation of neutrophil migration	9.20E-08	12
GO:0001932	regulation of protein phosphorylation	1.29E-06	64
GO:0032101	regulation of response to external stimulus	2.95E-18	82
GO:0080134	regulation of response to stress	8.03E-11	82
GO:0051046	regulation of secretion	1.65E-06	44
GO:0050863	regulation of T cell activation	3.00E-16	43
GO:0045580	regulation of T cell differentiation	8.30E-07	19
GO:0042129	regulation of T cell proliferation	1.47E-09	24
GO:0009617	response to bacterium	7.94E-14	61
GO:0009607	response to biotic stimulus	5.09E-16	100
GO:0034097	response to cytokine	2.46E-17	86
GO:0043207	response to external biotic stimulus	3.36E-16	99
GO:0032496	response to lipopolysaccharide	1.60E-09	33
GO:0002237	response to molecule of bacterial origin	1.60E-09	34
GO:0051707	response to other organism	3.19E-16	99
GO:0006979	response to oxidative stress	4.26E-07	35
GO:1901700	response to oxygen-containing compound	3.79E-08	85
GO:0046903	secretion	1.05E-22	111
GO:0032940	secretion by cell	3.41E-22	104
GO:0042110	T cell activation	6.40E-19	57
GO:0030217	T cell differentiation	1.07E-09	30
GO:0042098	T cell proliferation	1.93E-11	29
GO:0042330	taxis	5.14E-11	51
GO:0048771	tissue remodeling	1.71E-06	21
GO:0035295	tube development	7.48E-07	63
GO:0035239	tube morphogenesis	1.56E-06	54
GO:0001944	vasculature development	3.43E-07	50
GO:0006906	vesicle fusion	5.54E-20	80
GO:0099500	vesicle fusion to plasma membrane	1.95E-21	80
GO:0016050	vesicle organization	2.41E-19	110

Table S2. Gene lists for selected upregulated ontologies highly expressed in LT-HSCs sorted from WT→*Sost*$^{-/-}$ hematopoietic chimeras compared to LT-HSCs sorted from WT→WT hematopoietic chimeras

GO ID	Term	Gene names
GO:0002682	regulation of immune system process	Hfe Cebpb Mzb1 H2-DMb2 Dapk2 Oscar Blnk Ets1 Cd83 Adam8 Slc7a11 Vpreb3 Acod1 Havcr1 Pram1 Treml4 Cadm1 Fcer1a Fcer2a Cd101 Grap2 Lpxn Zc3h12d Slc9b2 Fes Ccl4 Ccl5 Ccr7 Gpnmb Mmp8 Mmp9 Mmp14 Hspa1a Hspa1b Alox15 Mpp1 Ikzf3 Pilra Anxa1 Fpr1 Fpr2 Id2 Btla Samhd1 Pparg Zap70 Arg2 Rhog Slc11a1 Csf3r Ighd Tnfsf13b Myo1f Prkcb Card11 Iglc1 Iglc2 Iglc3 Igll1 Il1b Scin Cd300e Il6 Il7r Ocstamp Cxcr2 Tarm1 Spi1 Inhba Spta1 Irf4 Dcstamp Itgal Il18rap Sit1 Itgb2 Vsir Ceacam1 Prdm1 Nod2 Blk Clec4e Pla2g2d Bst1 C1qc C3 C5ar1 Bank1 Rac2 Pla2g7 Rag1 Tnfaip8l2 Cxcr3 Gpr18 Rara Pglyrp1 Ighv1-18 Nlrx1 Cav1 Adtrp Nfam1 Ldlr Trdc Ifnlr1 Tfrc Rasgrp1 Thbs1 Cd2 Cd3d Cd3e Cd300ld Cd5 Cd8b1 Cd19 Ms4a1 Cd22 Cd80 Gpr68 Cd33 Cd37 Cd44 Trem1 Gpr137b Cd68 Serpinb1a Cd74 Cd79a Cd79b Lta Tnf Lef1 Trib1
GO:0030316	osteoclast differentiation	Cebpb Cd109 Oscar Tcirg1 Adam8 Slc9b2 Ccl5 Anxa2 Ocstamp Tnfrsf11a Dcstamp Tfrc Gpr68 Gpr137b Tnf
GO:0002274	myeloid leukocyte activation	Hexb Hk3 Abca13 Clec5a Dok3 Oscar Tcirg1 Chil1 Chit1 Adam8 Havcr1 S100a11 Pram1 Fcer1a Mgst1 Fes Ccl5 Plaur Mmp8 Aldh3b1 Mmp9 Hspa1a Hspa1b Alox5 Anxa2 Fpr1 Fpr2 Pparg Hvcn1 Degs1 Rhog Arsb Slc11a1 Slpi Myo1f Stx11 Il6 Ctsz Cxcr2 Cyba Tarm1 Galns Spi1 Gpr84 Irf4 Dcstamp Itgal Il18rap Itgb2 Ceacam1 Iqgap1 Blk Neu1 Bst1 Adgre4 Pygl C3 C5ar1 Ckap4 Rac2 Crispld2 Pglyrp1 Tom1 Lcn2 Nfam1 Olr1 Ldlr Gsn Mmp25 Rasgrp1 Thbs1 Cd2 Cd33 Cd37 Tlr1 Cd44 Gpr137b Cd53 Cd68 Serpinb1a Cd74 Tnf Mcemp1
GO:0030099	myeloid cell differentiation	Rasgrp4 Cebpb Cebpe Clec5a Cd109 Oscar Ets1 Tcirg1 Adam8 Klf2 Cd101 Slc9b2 Fes Ccl5 Ccr7 Mmp9 Hspa1a Hspa1b Anxa2 Id2 Pparg Csf3r Prkcb Scin Ocstamp Spi1 Inhba Irf4 Tnfrsf11a Dcstamp Ceacam1C1qc Rara Tfrc Thbs1 Gpr68 Gpr137bCd74 Tnf Lef1 Trib1

GO:0030098	lymphocyte differentiation	Blnk Tcirg1 Cd83 Adam8 Satb1 Ccr7 Mmp14 Ikzf3 Anxa1 Id2 Pou2af1 Zap70 Card11 Il1b Il6 Il7r Spi1 Inhba Ccr9 Irf4 Vsir Prdm1 Clec4e Gadd45g Pla2g2d Rag1 Gpr18 Rara Pglyrp1 Nfam1 Rasgrp1 Lig4 Cd2 Cd3d Cd3e Cd19 Ms4a1 Cd80 Cd44 Cd74 Cd79a Cd79b Lef1
GO:0002443	leukocyte mediated immunity	Hexb Hfe Hk3 Abca13 Clec5a Ncf1 Dok3 Oscar Tcirg1 Chil1 Chit1 Adam8 S100a11 Pram1 Cadm1 Fcer1a Fcer2a Mgst1 Fes Plaur Mmp8 Aldh3b1 Mmp9 Hspa1a Hspa1b Alox5 Anxa2 Fpr1 Fpr2 Hvcn1 Degs1 Rhog Arsb Slc11a1 Ighd Prf1 Slpi Myo1 Iglc1 Iglc2 Iglc3 Igll1 Il1b Stx11 Il6 Ctsz Il7r Cxcr2 Cyba Tarm1 Galns Gapt Gpr84 Itgal Ace Il18rap Itgb2 Vsir Ceacam1 Iqgap1 Nod2 Blk Neu1 Bst1 Adgre4 C1qc Pygl C3 C5ar1 Ckap4 Rac2 Crispld2 Pglyrp1 Ighv1-18 Tom1 Lcn2 Nfam1 Olr1 Trdc Gsn Tfrc Mmp25 Rasgrp1 Lig4 Cd19 Cd33 Cd44 Trem1 Cd53 Cd68 Serpinb1a Cd74 Lta Tnf Mcemp1
GO:0030098	lymphocyte differentiation	Blnk Tcirg1 Cd83 Adam8 Satb1 Ccr7 Mmp14 Ikzf3 Anxa1 Id2 Pou2af1 Zap70 Card11 Il1b Il6 Il7r Spi1 Inhba Ccr9 Irf4 Vsir Prdm1 Clec4e Gadd45g Pla2g2d Rag1 Gpr18 Rara Pglyrp1 Nfam1 Rasgrp1 Lig4 Cd2 Cd3d Cd3e Cd19 Ms4a1 Cd80 Cd44 Cd74 Cd79a Cd79b Lef1
GO:0071887	leukocyte apoptotic process	Adam8 Slc7a11 Ccl5 Ccr7 Anxa1 St6gal1 Arg2 Il6 Il7r Cxcr2 Nod2 Blk Rag1 Cd44 Cd74 Fcmr
GO:0045321	leukocyte activation	Hexb Hfe Cebpb Hk3 Mzb1 H2-DMb2 Abca13 Clec5a Dok3 Oscar Blnk Tcirg1 Cd83 Chil1 Chit1 Adam8 Havcr1 S100a11 Pram1 Cadm1 Fcer1a Satb1 Mgst1 Grap2 Zc3h12d Fes Ccl5 Plaur Ccr7 Gpnmb Mmp8 Aldh3b1 Mmp9 Mmp14 Hspa1a Hspa1b Alox5 Ikzf3 Anxa1 Anxa2 Fpr1 Fpr2 Id2 Pou2af1 Btla Pparg Hvcn1 Zap70 Degs1 Arg2 Rhog Arsb Slc11a1 Ighd Prf1 Tnfsf13b Slpi Myo1f Prkcb Card11 Iglc1 Iglc2 Iglc3 Igll1 Il1b Stx11 Il6 Ctsz Il7r Cxcr2 Cyba Tarm1 Galns Spi1 Inhba Ccr9 Spta1 Gapt Gpr84 Irf4 Dcstamp Itgal Il18rap Sit1 Itgb2 Vsir Ceacam1 Iqgap1 Prdm1 Nod2 Blk Cxcr5 Clec4e Neu1 Gadd45g Pla2g2d Bst1 Adgre4 Pygl C3 C5ar1 Ckap4 Bank1 Rac2 Crispld2 Rag1 Tnfaip8l2 Gpr18 Rara Pglyrp1 Ighv1-18 Tom1 Cav1 Lcn2 Nfam1 Olr1 Ldlr Trdc Gsn Tfrc Mmp25 Rasgrp1 Lig4 Thbs1 Cd2 Cd3d Cd3e Cd5 Cd8b1 Cd19 Ms4a1 Cd22 Cd80 Cd33 Cd37 Tlr1 Cd44 Gpr137b Cd53 Cd68 Serpinb1a Cd74 Cd79a Cd79b Tnf Lef1 Mcemp1

GO:0006954	inflammatory response	Hfe Pik3ap1 Pde2a Cebpb Acp5 Ncf1 Blnk Ets1 Tcirg1 Chil1 Adam8 Fabp4 Acod1 Fcer1a Mgst2 Ccl4 Ccl5 Ccr3 Ccr7 Ltb4r1 Mmp8 Mmp9 Prdx5 Plp1 Sdc1 Alox5 Alox15 Hyal1 Anxa1 Fpr1 Fpr2 Aoah Pparg Slc11a1 Il1b Mapk13 Il6 Cxcr2 Cyba Tarm1 Nlrp12 Spp1 Tnfrsf11a Itgal Ace Il18rap Itgb2 Ptgir Ffar4 Nod2 Pla2g2d Il1r2 Bst1 C3 C5ar1 Pla2g7 Tnfaip8l2 Cxcr3 Pglyrp1 Nt5e Nlrx1 Akna Pstpip1 Nfam1 Cxcl2 Olr1 Ldlr Mmp25 Rasgrp1 Osm Thbs1 Gpr68 Tlr1 Cd44 Trem1 Cd68 Serpinb1a Lta Tnf
GO:0030097	hemopoiesis	Rasgrp4 Cebpb Cebpd Cebpe Arl11 Clec5a Cd109 Oscar Blnk Ets1 Tcirg1 Cd83 Adam8 Klf2 Satb1 Cd101 Slc9b2 Fes Ccl5 Ccr7 Mmp9 Mmp14 Rbm47 Hspa1a Hspa1b Ikzf3 Anxa1 Anxa2 Id2 Pou2af1 Pparg Zap70 Csf3r Prkcb Card11 Il1b Scin Il6 Il7r Ocstamp Spi1 Inhba Ccr9 Spta1 Sptb Irf4 Tnfrsf11a Dcstamp Ace Vsir Ceacam1 Prdm1 Blk Clec4e Gadd45g Pla2g2d C1qc Rag1 Rogdi Gpr18 Rara Pglyrp1 Nfam1 Tfrc Rasgrp1 Lig4 Osm Thbs1 Cd2 Cd3d Cd3e Cd19 Ms4a1 Cd80 Gpr68 Cd44 Gpr137b Cd74 Cd79a Cd79b Tnf Lef1 Trib1
GO:0006952	defense response	Hfe Pik3ap1 Pde2a Cebpb Cebpe Clec5a Acp5 Ncf1 Blnk Ets1 Tcirg1 Cd83 Chil1 Slc15a3 Adam8 Fabp4 Acod1 Treml4 Cadm1 Fcer1a Mgst2 Fes Ifi30 Ccl4 Ccl5 Ccr3 Ccr7 Ltb4r1 Mmp8 Mmp9 Prdx5 Plp1 Sdc1 Alox5 Alox15 Hyal1 Anxa1 Fpr1 Fpr2 Aoah Samhd1 Pparg Zap70 Arg2 Mst1r Slc11a1 Csf3r Ighd Prf1 Slpi Myo1f Card11 Iglc1 Iglc2 Iglc3 Igll1 Il1b Mapk13 Stx11 Cd300e Il6 Il7r Cxcr2 Cyba Tarm1 Nlrp12 Msrb1 Inhba Spp1 Ccr9 Irf4 Ncf2 Tnfrsf11a Itgal Ace Il18rap Itgb2 Ptgir Ffar4 Ceacam1 Prdm1 Nod2 Blk Clec4e Pla2g2d Il1r2 Bst1 Kcnj8 C1qc C3 C5ar1 Pla2g7 Rag1 Tnfaip8l2 Cxcr3 Pglyrp1 Nt5e Ighv1-18 Nlrx1 Akna Oas3 Pstpip1 Lcn2 Nfam1 Cxcl2 Olr1 Ldlr Trdc Cldn1 Ifnlr1 Gsn Mmp25 Rasgrp1 Osm Thbs1 Gpr68 Cd37 Tlr1 Cd44 Trem1 Cd68 Serpinb1a Cd74 Irgc1 Lsp1 Lta Tnf Fcmr
GO:0045453	bone resorption	Acp5 Oscar Tcirg1 Adam8 Ctsk Il6 Spp1 Tnfrsf11a Dcstamp Plekhm1 Rac2 Tfrc Gpr137b

Table S3. Complete blood count values

Genotype & Sex		WBC K/uL	Ne# K/uL	Ly# K/uL	Mo# K/uL	Eo# K/uL	Ba# K/uL	Ne% %	Ly% %	Mo% %	Eo% %	Ba% %	RBC M/uL	HB g/dL	HCT %	MCV fL	MCH Pg	MCHC g/dL	RDW %	PLT K/uL	MPV fL
	control	*17.32*	*9.83*	*6.15*	*0.39*	*0.16*	*0.79*	*56.73*	*36*	*2.26*	*0.95*	*4.55*	*9.09*	*16.3*	*52.6*	*57.9*	*17.9*	*31*	*26.1*	*967*	*7.6*
**WT Female**	1	7.16	1.34	5.54	0.22	0.04	0.01	18.72	77.4	3.12	0.57	0.16	9.97	13.8	53.3	53.5	13.8	25.9	16.5	802	4.2
	2	9.02	1.21	7.12	0.58	0.09	0.02	13.44	78.9	6.38	1.04	0.22	10.18	14.4	53.1	52.2	14.1	27.1	17.4	739	4.4
	3	9.16	0.76	7.64	0.74	0.02	0.01	8.29	83.4	8.07	0.19	0.07	10.5	14.8	56.5	53.8	14.1	26.2	16.5	695	4.3
	4	10	1.17	8.27	0.51	0.03	0.01	11.73	82.7	5.09	0.32	0.13	10.8	14.7	55.6	51.5	13.6	26.4	17.2	614	4.4
	5	10.14	1.1	8.46	0.45	0.1	0.03	10.86	83.4	4.42	0.99	0.29	9.78	14.5	51.9	53.1	14.8	27.9	17.1	642	4.3
	**Mean**	**9.096**	**1.12**	**7.406**	**0.5**	**0.06**	**0.02**	**12.61**	**81.2**	**5.42**	**0.62**	**0.17**	**10.25**	**14.4**	**54.1**	**52.8**	**14.1**	**26.7**	**16.9**	**698**	**4.32**
	**SD**	**1.19**	**0.22**	**1.17**	**0.19**	**0.04**	**0.01**	**3.89**	**2.81**	**1.89**	**0.38**	**0.08**	**0.409**	**0.39**	**1.9**	**0.95**	**0.45**	**0.803**	**0.42**	**75.3**	**0.08**
***Sost*$^{-/-}$ Female**	11	7.28	1.66	5.09	0.46	0.06	0.01	22.76	69.9	6.34	0.81	0.17	9.44	13.4	53.1	56.2	14.2	25.2	16.5	659	4.7
	12	5	0.27	4.34	0.32	0.04	0.02	5.47	86.9	6.35	0.79	0.5	8.72	13.3	47.4	54.4	15.3	28.1	18	549	4.7
	13	7.68	0.93	6.27	0.38	0.07	0.02	12.15	81.7	4.92	0.94	0.3	9.21	13.9	50.5	54.8	15.1	27.5	17.8	607	4.7
	14	4.26	0.49	3.26	0.46	0.03	0.01	11.6	76.5	10.9	0.82	0.23	8.77	14.2	47.8	54.5	16.2	29.7	18.8	606	5
	15	6.42	0.77	5.03	0.52	0.08	0.02	12.02	78.3	8.09	1.3	0.3	10.25	14.1	58.2	56.8	13.8	24.2	16.8	570	4.6
	**Mean**	**6.128**	**0.82**	**4.798**	**0.43**	**0.06**	**0.02**	**12.8**	**78.6**	**7.32**	**0.93**	**0.3**	**9.278**	**13.8**	**51.4**	**55.3**	**14.9**	**26.94**	**17.6**	**598**	**4.74**
	**SD**	**1.465**	**0.53**	**1.105**	**0.08**	**0.02**	**0.01**	**6.233**	**6.29**	**2.3**	**0.21**	**0.12**	**0.622**	**0.41**	**4.44**	**1.09**	**0.95**	**2.226**	**0.93**	**42**	**0.15**
**WT Male**	6	18.04	4.2	13.33	0.39	0.08	0.04	23.29	73.9	2.16	0.45	0.2	10.27	14.2	52.8	51.4	13.8	26.9	18.1	927	4.3
	7	16.38	2.57	13.15	0.57	0.07	0.01	15.72	80.3	3.5	0.42	0.09	10.14	13.7	52.5	51.8	13.5	26.1	17.5	887	4.3
	8	16.86	2.27	13.72	0.74	0.1	0.03	13.44	81.4	4.4	0.6	0.15	10.12	13.7	51.5	50.9	13.5	26.6	16.9	817	4.2
	9	17.98	2.09	14.9	0.91	0.07	0.01	11.61	82.9	5.05	0.4	0.07	10.86	14.9	57.2	52.7	13.7	26	17.3	746	4.1
	10	11.02	0.41	10.04	0.51	0.04	0.02	3.76	91.1	4.61	0.34	0.17	5.65	7.6	29	51.3	13.5	26.2	16.3	503	4.2
	**Mean**	**16.06**	**2.31**	**13.03**	**0.62**	**0.07**	**0.02**	**13.56**	**81.9**	**3.94**	**0.44**	**0.14**	**9.408**	**12.8**	**48.6**	**51.6**	**13.6**	**26.36**	**17.2**	**776**	**4.22**
	**SD**	**2.905**	**1.35**	**1.804**	**0.2**	**0.02**	**0.01**	**7.057**	**6.18**	**1.15**	**0.1**	**0.05**	**2.122**	**2.96**	**11.2**	**0.68**	**0.14**	**0.378**	**0.67**	**168**	**0.08**
***Sost*$^{-/-}$ Male**	16	11	1.06	9.2	0.65	0.07	0.02	9.61	83.6	5.93	0.65	0.2	9.47	13.2	51.7	54.6	13.9	25.5	17.8	774	4.7
	17	11.62	1.02	9.78	0.72	0.08	0.02	8.76	84.1	6.23	0.69	0.18	9.8	13.8	53.7	54.8	14.1	25.7	17.3	640	4.3
	18	11.96	1.38	9.58	0.86	0.09	0.04	11.56	80.1	7.2	0.78	0.35	9.09	13.9	47.6	52.4	15.3	29.2	18.1	782	4.5
	19	10.44	0.57	9.15	0.68	0.02	0.02	5.5	87.7	6.52	0.16	0.15	9.14	13.4	49.6	54.3	14.7	27	19.5	643	4.4
	20	12.24	0.81	10.62	0.79	0.02	0	6.62	86.8	6.42	0.18	0.04	9.28	13.4	49.3	53.1	14.4	27.2	18.6	627	4.5
	**Mean**	**11.45**	**0.97**	**9.666**	**0.74**	**0.06**	**0.02**	**8.41**	**84.5**	**6.46**	**0.49**	**0.18**	**9.356**	**13.5**	**50.4**	**53.8**	**14.5**	**26.92**	**18.3**	**693**	**4.48**
	**SD**	**0.731**	**0.3**	**0.595**	**0.09**	**0.03**	**0.01**	**2.406**	**2.97**	**0.47**	**0.3**	**0.11**	**0.289**	**0.3**	**2.36**	**1.04**	**0.55**	**1.482**	**0.84**	**77.7**	**0.15**

*Highlighted means are to show where WT and *Sost*$^{-/-}$ are statistically different, compared by sex, using students ttest $p<0.05$

Table S4. Antibodies used for flow cytometry

Specificity	Clone	Fluorochrome
Annexin V		APC
Annexin V		FITC
7AAD		
CD11b	M1/70	Biotin
CD127 (Il7rα)	A7R34	Biotin
CD127 (Il7rα)	A7R34	PerCP Cy5.5
CD150	mShad	FITC
CD16/32	93	PerCP-Cy5.5
CD19	6D5	Biotin
CD19	6D5	PE
CD3	2C11	Biotin
CD3	2C11	APC
CD31	390	APC
CD34	RAM34	FITC
CD4	Gk1.5	Biotin
CD41	MWReg30	BUV395
CD45	30-F11	APC-Cy7
CD45	30-F11	PerCP Cy5.5
CD45.1	A20	FITC
CD45.1	A20	PE
CD45.1	A20	PerCP-Cy5.5
CD45.2	104	APC-Cy7
CD45R (B220)	RA3-6B2	Biotin
CD48	HM48	PE-Cy7
CD48	HM48	APC
CD5	53-7.3	Biotin
CD51	RMV-7	Biotin
CD8	53.6.7	Biotin
cKit	2B8	APC
cKit	2B8	APC
F4/80	BM8	Biotin
Flk2	A2F10	PE
Gr1	RB6-8C3	Biotin
Gr1	RB6-8C3	PE-Cy7
Ki67	SolA15	APC
NK1.1	PK136	Biotin
Sca1	D7	BV510
Sca1	D7	FITC
Streptavidin		PerCP Cy5.5
Streptavidin		PacBlue
Streptavidin		PE
Ter119	TER119	Biotin
Ter119	TER119	BV421

Table S5. List of Cell Surface Markers used for Flow Cytometry

Cell Type	Markers				
Transplanted LT-HSCs	$Lin^- Sca1^+ cKit^+$ (LSK)	$CD150^+$	$CD48^-$		
All other LT-HSC	$Lin^- Sca1^+ cKit^+$ (LSK)	$CD150^+$	$CD48^-$	$Flk2^-$	
ST-HSCs	$Lin^- Sca1^+ cKit^+$ (LSK)	$CD150^-$	$CD48^-$	$Flk2^-$	
MPP2	$Lin^- Sca1^+ cKit^+$ (LSK)	$CD150^+$	$CD48^+$	$Flk2^-$	
MPP3	$Lin^- Sca1^+ cKit^+$ (LSK)	$CD150^-$	$CD48^+$	$Flk2^-$	
MPP4	$Lin^- Sca1^+ cKit^+$ (LSK)	$CD150^-$	$CD48^+$	$Flk2^+$	
T Cells	$Gr1^-$	$CD3^+$	$CD19^-$		
B Cells	$Gr1^-$	$CD3^-$	$CD19^+$		
Monocytes	$CD3^-$	$CD11b^+$	$Gr1^-$		
Granulocytes	$CD3^-$	$CD11b^+$	$Gr1^+$		
B Cell, Fraction A (BM)	$B220^+ CD43^+$	$cKit^-$	$CD19^-$		
B Cell, Fraction B-C (BM)	$B220^+ CD43^+$	$cKit^-$	$CD19^+$		
B Cell, Fraction D (BM)	$B220^+ CD43^-$	$cKit^-$	$CD19^+$	IgM^-	IgD^-
B Cell, Fraction E (BM)	$B220^+ CD43^-$	$cKit^-$	$CD19^+$	IgM^+	IgD^-
B Cell, Fraction F (BM)	$B220^+ CD43^-$	$cKit^-$	$CD19^+$	IgD^+	
B Cell (Spleen)	$B220^+$	$CD19^+$			
B Cell (Spleen)	$B220^+$	IgM^+			
Mesenchymal stromal cell	Lin^-, $CD45^-$	$CD31^-$	$Sca1^+$	$CD51^+$	
Osteoblasts	Lin^-, $CD45^-$	$CD31^-$	$Sca1^-$	$CD51^+$	
Endothelial cell	Lin^-, $CD45^-$	$CD31^+$	$Sca1^+$		
"Other" Bone cell	Lin^-, $CD45^-$	$CD31^-$	$Sca1^-$	$CD51^-$	

Supplemental Figure Legends

Figure S1. Sclerostin-depleting antibodies change hematopoietic differentiation. (A) Bone marrow cellularity of HSPCs in PBS vs Scl-Ab treatment (B) Bone marrow cellularity of mature lineages of PBS vs Scl-Ab treatment (CS) Splenic cellularity of HSPCs in PBS vs Scl-Ab treatment (D) Splenic cellularity of mature lineages of PBS vs Scl-Ab treatment (E) Bone marrow frequency of B cell populations (F) Splenic frequency of B cell populations (G) Bone marrow cellularity of B cell populations (H) Splenic cellularity of B cell populations Mice were 8 weeks of age at start of treatment, female C57B6. 7 mice received PBS, 7 mice received Scl-Ab antibody.

Figure S2. Analysis of LT-HSC frequency and cellularity in Sost$^{-/-}$ mice. (A) Bone marrow frequency of HSPCs in WT and *Sost*$^{-/-}$ mice (B) Bone marrow cellularity of HSPCs in *Sost*$^{-/-}$ mice (C) Splenic frequency of HSPCs in WT and *Sost*$^{-/-}$ mice (D) Splenic cellularity of HSPCs in *Sost*$^{-/-}$ mice. Mice ages ranged from 14-40 weeks of age, with equal male and female numbers for each age (2-4 mice per sample and time point).

Figure S3. Proliferation and cell death are altered in Sost$^{-/-}$ bone marrow and spleens. (A) Donor cellularity of BM HSPC progenitors in WT→*Sost*$^{-/-}$ chimeras (B) Donor cellularity of spleen HSPC progenitors in WT→*Sost*$^{-/-}$ chimeras. 5FU analysis consisted of 1 experiment (C) Gene expression of cell cycle genes in LSKs normalized to GAPDH (D) Cell numbers in controls and 5-FU treated animals in 2 independent experiments 5 days after 5FU injection (E) Summary of basal (non-5FU-treated mice) cell cycle analysis in LSKs (F) Summary of cell cycle analysis in WT and *Sost*$^{-/-}$ mice in LSKs 5 days after 5-FU injection. LSK frequencies in individual mice are shown as normalized to the mean of the WT 5-FU treated mice. (G) Representative flow cytometry plots of cell cycle analysis on gated LSKs in untreated and 5FU-treated animals. Mice were 17 weeks of age, male and female.

Figure S4. Hematopoietic progenitors derived from Sost$^{-/-}$ bone marrow display normal hematopoiesis after transplantation into a WT microenvironment. WT (CD45.2)→ WT (CD45.1)(control, black bars) and *Sost*$^{-/-}$ (CD45.2)→ WT (CD45.1) (teal bars) hematopoietic chimeras were prepared to test the function of hematopoietic progenitors derived from *Sost-/-* bone marrow donors after transplantation into WT recipients. (A) Percent donor hematopoietic chimerism; (B) Total bone marrow cellularity; (C) Frequency of donor-derived BM LSKs; (D) Frequency of donor-derived splenic LSKs; (E) Frequency of donor-derived BM HSPCs; (F) Frequency of donor-derived splenic HSPCs. The ages of the WT donor mice ranged from 12-15 weeks, ages of *Sost*$^{-/-}$donor mice ranged from 12-17 weeks. Ages of the recipients ranged from 13-15 weeks. Both male and female donors and hosts were used. Analysis of BM was performed 19-20 weeks post-

transplantation. Data shown are compiled from 3 independent experiments. No statistical differences between WT→WT and *Sost*$^{-/-}$→WT chimeras were found ($p<0.05$, Mann-Whitney, non-parametric t-test).

Figure S5. RNA-Seq heatmap results. Osteoclast differentiation and bone resorption genes and inflammation genes upregulated in sorted WT LT-HSCs developing in *Sost*-/- bone marrow microenvironments (WT→S*ost*-/- bone marrow chimeras).

Figure S6. ScRNA-Seq Analysis of immune cells from the long bones from WT and SostKO mice. (A) Heatmap showing the expression of top markers of each immune cell clusters as defined in Figure 4 of the manuscript; (B) Feature plots showing the expression of selected cluster markers and *Ptprc* (CD45).

Figure S7. Analysis of CXCL12 and SCF in bone stromal cells of Sost$^{-/-}$ *mice.* (A) Representative FACS plots depicting gating strategy for collagenase-digested bones (B) Relative CXCL12 gene expression in *Sost*$^{-/-}$ bone cells (C) Relative SCF gene expression in *Sost*$^{-/-}$ bone cells (D) Summary of bone cell frequencies after digest of femurs and tibiae of WT (n=14) and KO (n=26) mice. $p<0.01$** Mann-Whitney, non-parametric ttest.

Figure S8. Evidence of extramedullary hematopoiesis in Sost$^{-/-}$ *mice.* (A) Spleen weight in WT and *Sost*$^{-/-}$ mice. Values represent total spleen weight divided by total mouse weight (B) Splenic pulp area, as calculated by area of indicated pulp type (white or red) divided by total pulp area stained. (C) H&E staining of spleens. Size reference is 500 μm. Dark purple round spots are white pulp and pink area is red pulp. (D-G) Data is representative of 4 mice per time point for WT and *Sost*$^{-/-}$ mice. (D) LT-HSC frequency analyzed over time in the bone marrow (E) ST-HSC frequency analyzed over time in the bone marrow (F) LT-HSC frequency analyzed over time in the spleen (G) ST-HSC frequency analyzed over time in the spleen. Mice ages ranged from 14-40 weeks of age, with equal male and female numbers for each age (2-4 mice per sample and time point). $p<0.05$*, $p<0.01$**, $p<0.001$*** Mann-Whitney, non-parametric ttest.

Figure S9. LT-HSCs from the spleen show no functional differences compared to the bone marrow. (A) Experimental schematic of splenic LT-HSCs transplants (B) Bone marrow and splenic cellularity of host transplants (C) Bone marrow and splenic donor chimerism from transplants (D) Bone marrow cellularity of HSPCs from donor (E) Splenic cellularity of HSPCs from donor (F) Bone marrow cellularity of mature lineages from donor (G) Splenic cellularity of mature lineages from donor. Data were collected in 2 separate experiments. Donor ages ranged from 37-49 weeks male or female. Host ages ranged from 11-15 weeks male and female. Transplant time was 19 weeks. Donor LT-HSC cell numbers ranged from 1,000-3,000 LT-HSCs cells per mouse.

Supplemental References

1. Schepers, K., E. C. Hsiao, T. Garg, M. J. Scott, and E. Passegue. 2012. Activated Gs signaling in osteoblastic cells alters the hematopoietic stem cell niche in mice. *Blood* 120: 3425-3435.
2. Livak, K. J., and T. D. Schmittgen. 2001. Analysis of relative gene expression data using real-time quantitative PCR and the 2(-Delta Delta C(T)) Method. *Methods* 25: 402-408.
3. Cain, C. J., R. Rueda, B. McLelland, N. M. Collette, G. G. Loots, and J. O. Manilay. 2012. Absence of sclerostin adversely affects B-cell survival. *J Bone Miner Res* 27: 1451-1461.

Figure S1

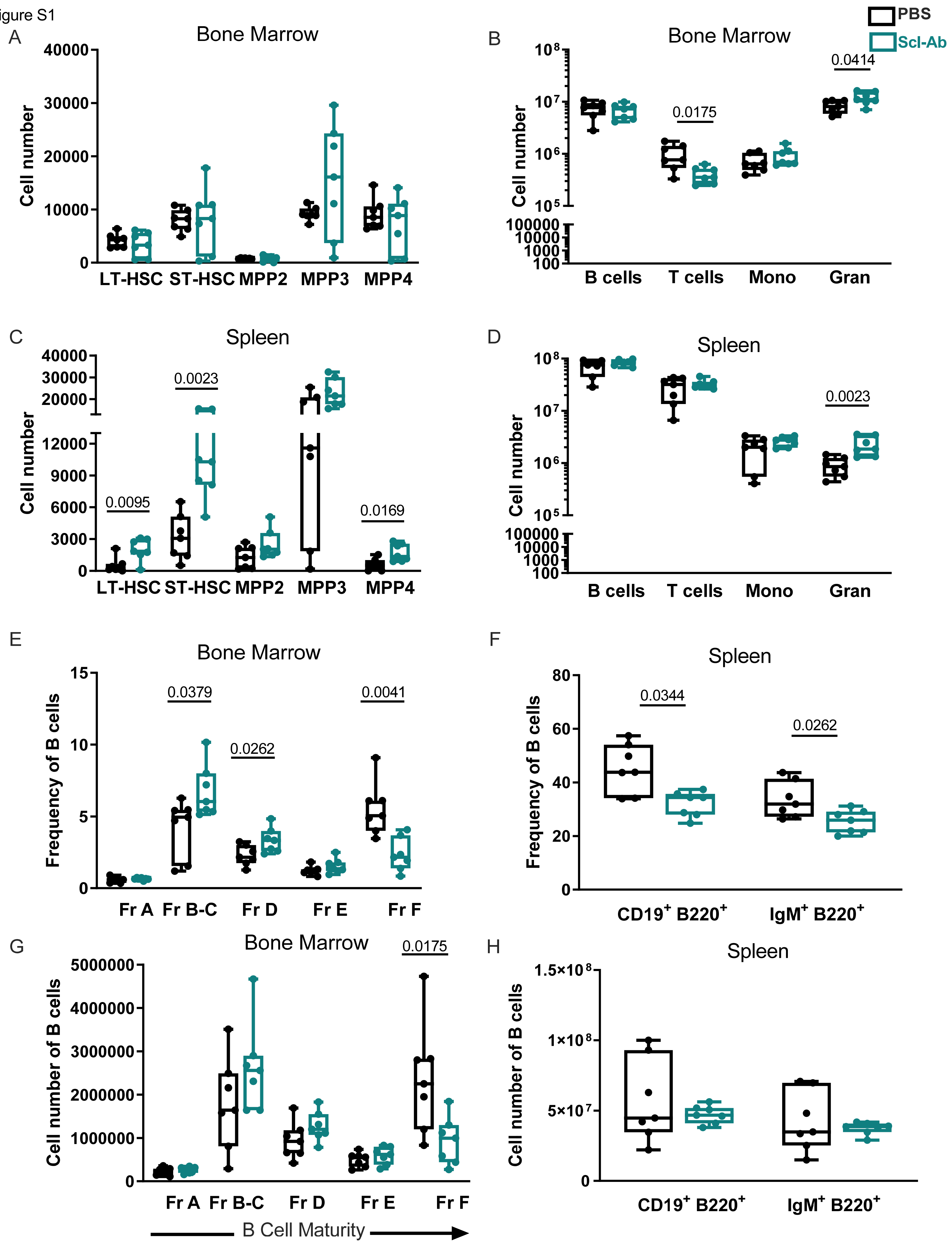
PBS
Scl-Ab
A
Bone Marrow
Cell number
LT-HSC ST-HSC MPP2 MPP3 MPP4
B
Bone Marrow
0.0175
0.0414
B cells T cells Mono Gran
C
Spleen
0.0095
0.0023
0.0169
D
Spleen
0.0023
E
Bone Marrow
Frequency of B cells
0.0379
0.0262
0.0041
Fr A Fr B-C Fr D Fr E Fr F
F
Spleen
0.0344
0.0262
CD19+ B220+
IgM+ B220+
G
Bone Marrow
0.0175
Cell number of B cells
B Cell Maturity
H
Spleen

Figure S2

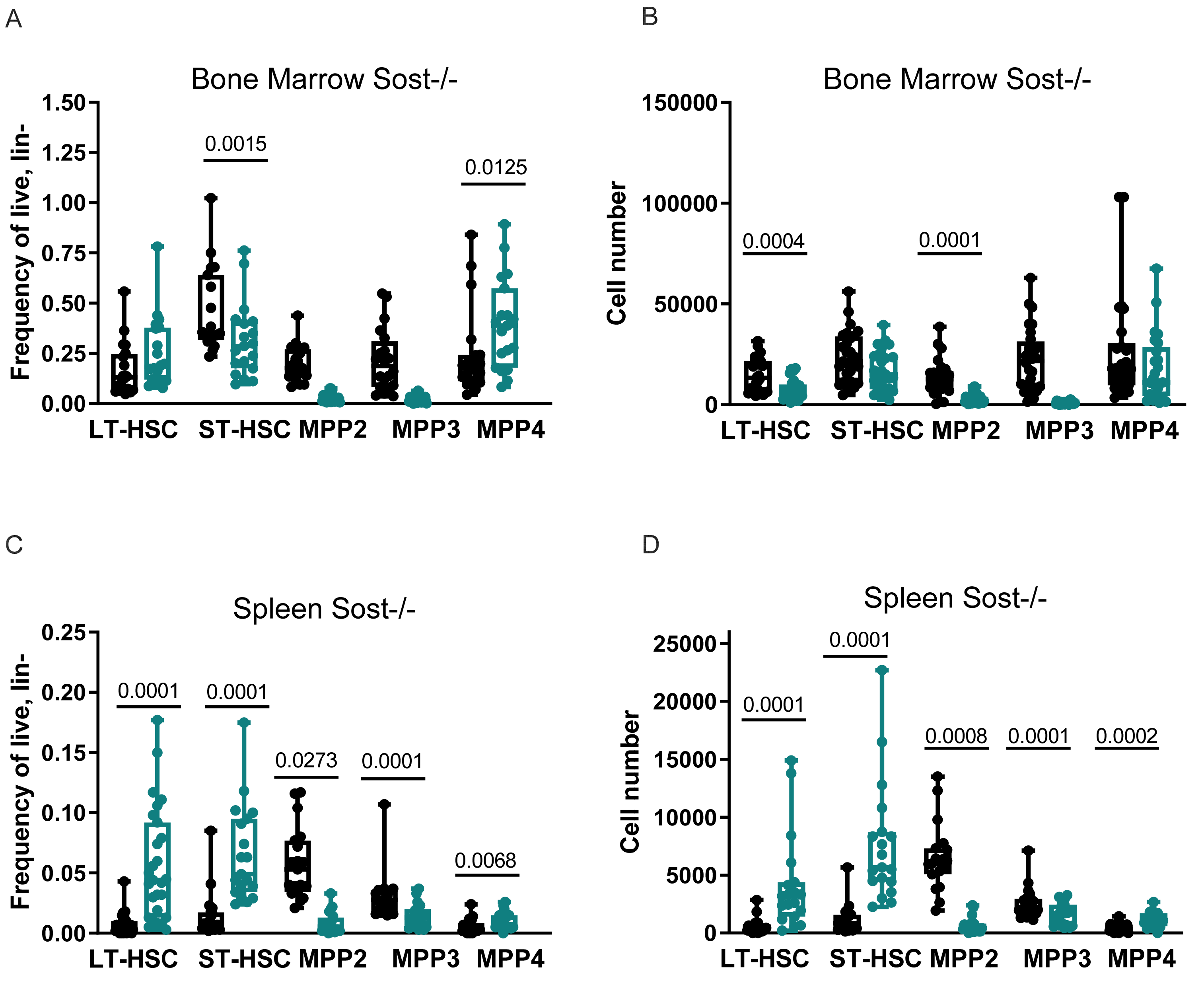
A
Bone Marrow Sost-/-
Frequency of live, lin-
1.50
1.25
1.00
0.75
0.50
0.25
0.00
0.0015
0.0125
LT-HSC
ST-HSC
MPP2
MPP3
MPP4
B
Bone Marrow Sost-/-
Cell number
150000
100000
50000
0
0.0004
0.0001
LT-HSC
ST-HSC
MPP2
MPP3
MPP4
C
Spleen Sost-/-
Frequency of live, lin-
0.25
0.20
0.15
0.10
0.05
0.00
0.0001
0.0001
0.0273
0.0001
0.0068
LT-HSC
ST-HSC
MPP2
MPP3
MPP4
D
Spleen Sost-/-
Cell number
25000
20000
15000
10000
5000
0
0.0001
0.0001
0.0008
0.0001
0.0002
LT-HSC
ST-HSC
MPP2
MPP3
MPP4

Figure S3

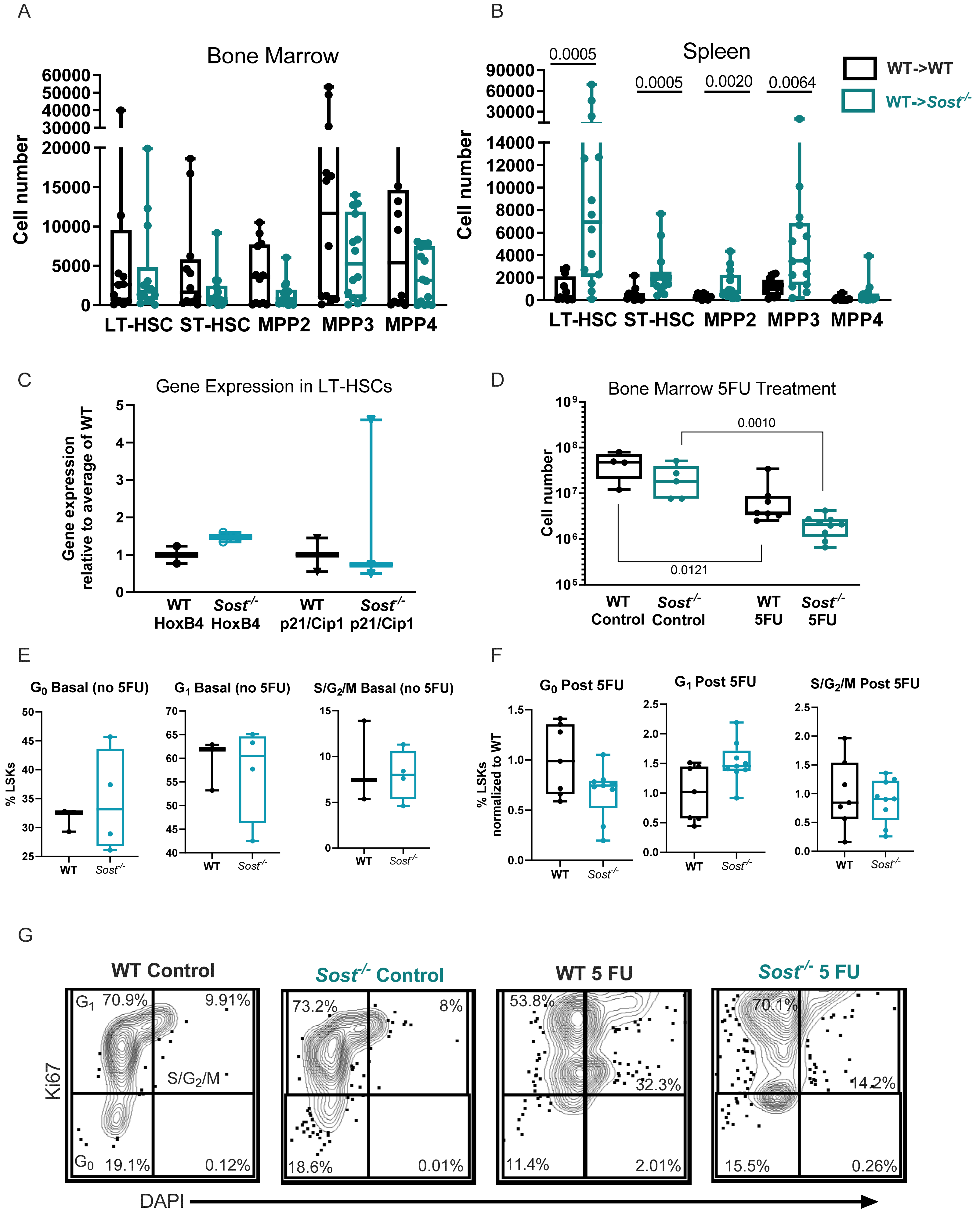
A
Bone Marrow
Cell number
LT-HSC ST-HSC MPP2 MPP3 MPP4
B
Spleen
0.0005
0.0005
0.0020
0.0064
WT->WT
WT->Sost-/-
LT-HSC ST-HSC MPP2 MPP3 MPP4
C
Gene Expression in LT-HSCs
Gene expression relative to average of WT
WT HoxB4
Sost-/- HoxB4
WT p21/Cip1
Sost-/- p21/Cip1
D
Bone Marrow 5FU Treatment
0.0010
0.0121
WT Control
Sost-/- Control
WT 5FU
Sost-/- 5FU
E
G0 Basal (no 5FU)
G1 Basal (no 5FU)
S/G2/M Basal (no 5FU)
% LSKs
WT
Sost-/-
F
G0 Post 5FU
G1 Post 5FU
S/G2/M Post 5FU
% LSKs normalized to WT
G
WT Control
Sost-/- Control
WT 5 FU
Sost-/- 5 FU
Ki67
DAPI
G1 70.9%
9.91%
S/G2/M
G0 19.1%
0.12%
73.2%
8%
18.6%
0.01%
53.8%
32.3%
11.4%
2.01%
70.1%
14.2%
15.5%
0.26%

Figure S4

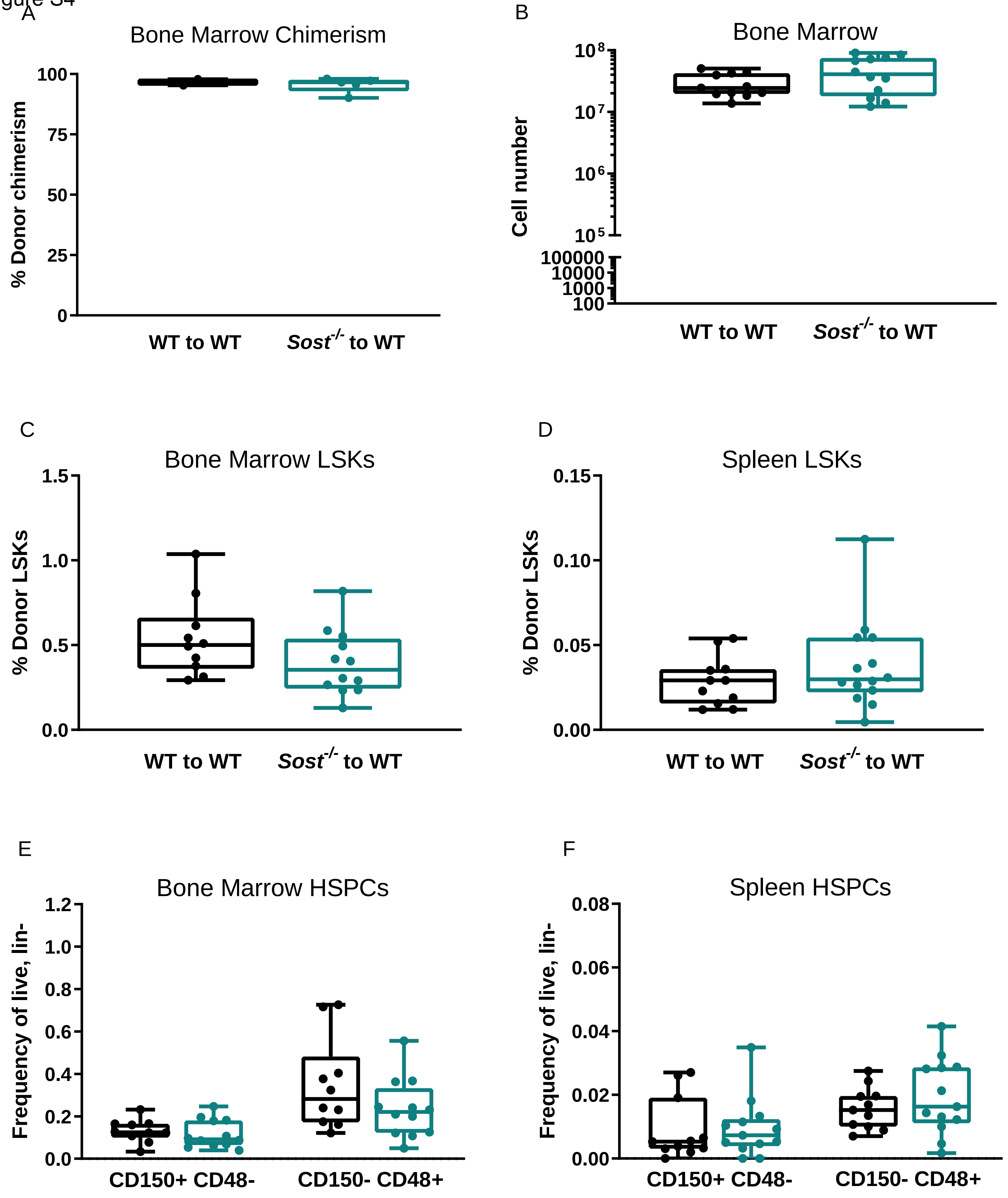
A
Bone Marrow Chimerism
% Donor chimerism
100
75
50
25
0
WT to WT
Sost-/- to WT
B
Bone Marrow
Cell number
10^8
10^7
10^6
10^5
100000
10000
1000
100
WT to WT
Sost-/- to WT
C
Bone Marrow LSKs
% Donor LSKs
1.5
1.0
0.5
0.0
WT to WT
Sost-/- to WT
D
Spleen LSKs
% Donor LSKs
0.15
0.10
0.05
0.00
WT to WT
Sost-/- to WT
E
Bone Marrow HSPCs
Frequency of live, lin-
1.2
1.0
0.8
0.6
0.4
0.2
0.0
CD150+ CD48-
CD150- CD48+
F
Spleen HSPCs
Frequency of live, lin-
0.08
0.06
0.04
0.02
0.00
CD150+ CD48-
CD150- CD48+

Figure S5

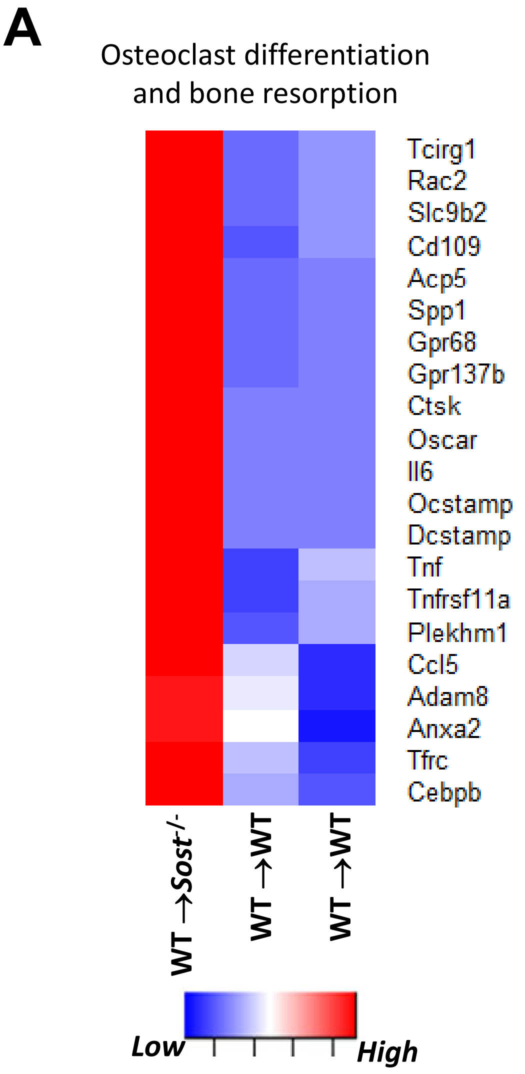
A
Osteoclast differentiation and bone resorption
Tcirg1
Rac2
Slc9b2
Cd109
Acp5
Spp1
Gpr68
Gpr137b
Ctsk
Oscar
Il6
Ocstamp
Dcstamp
Tnf
Tnfrsf11a
Plekhm1
Ccl5
Adam8
Anxa2
Tfrc
Cebpb
WT →Sost-/-
WT →WT
WT →WT
Low
High

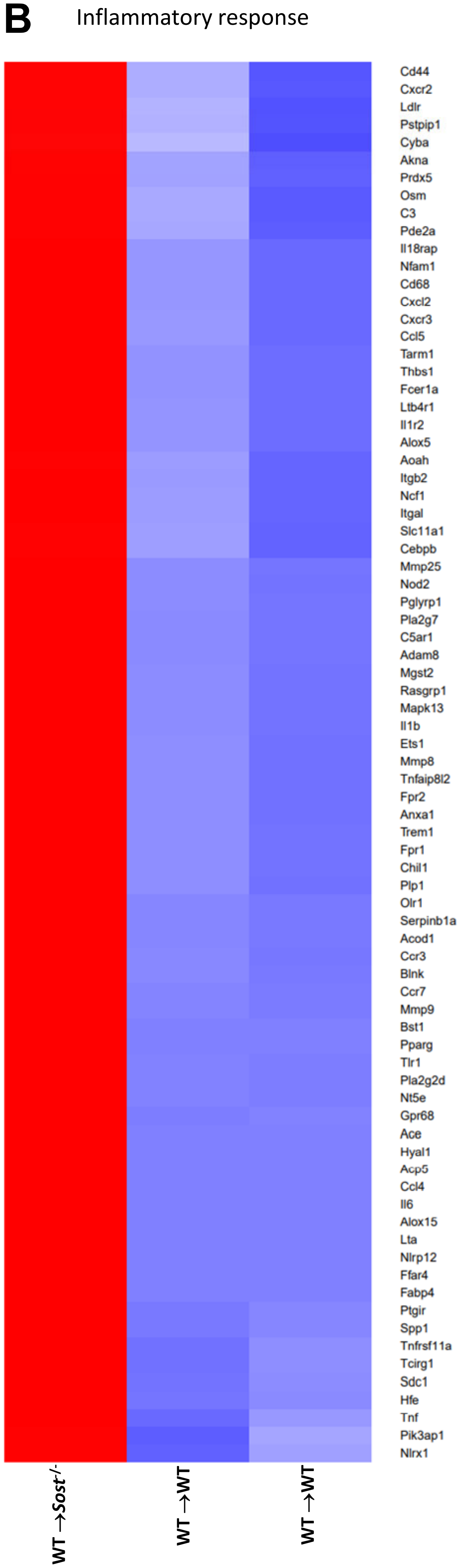
B
Inflammatory response
Cd44
Cxcr2
Ldlr
Pstpip1
Cyba
Akna
Prdx5
Osm
C3
Pde2a
Il18rap
Nfam1
Cd68
Cxcl2
Cxcr3
Ccl5
Tarm1
Thbs1
Fcer1a
Ltb4r1
Il1r2
Alox5
Aoah
Itgb2
Ncf1
Itgal
Slc11a1
Cebpb
Mmp25
Nod2
Pglyrp1
Pla2g7
C5ar1
Adam8
Mgst2
Rasgrp1
Mapk13
Il1b
Ets1
Mmp8
Tnfaip8l2
Fpr2
Anxa1
Trem1
Fpr1
Chil1
Plp1
Olr1
Serpinb1a
Acod1
Ccr3
Blnk
Ccr7
Mmp9
Bst1
Pparg
Tlr1
Pla2g2d
Nt5e
Gpr68
Ace
Hyal1
Acp5
Ccl4
Il6
Alox15
Lta
Nlrp12
Ffar4
Fabp4
Ptgir
Spp1
Tnfrsf11a
Tcirg1
Sdc1
Hfe
Tnf
Pik3ap1
Nlrx1
WT →Sost-/-
WT →WT
WT →WT

Figure S6

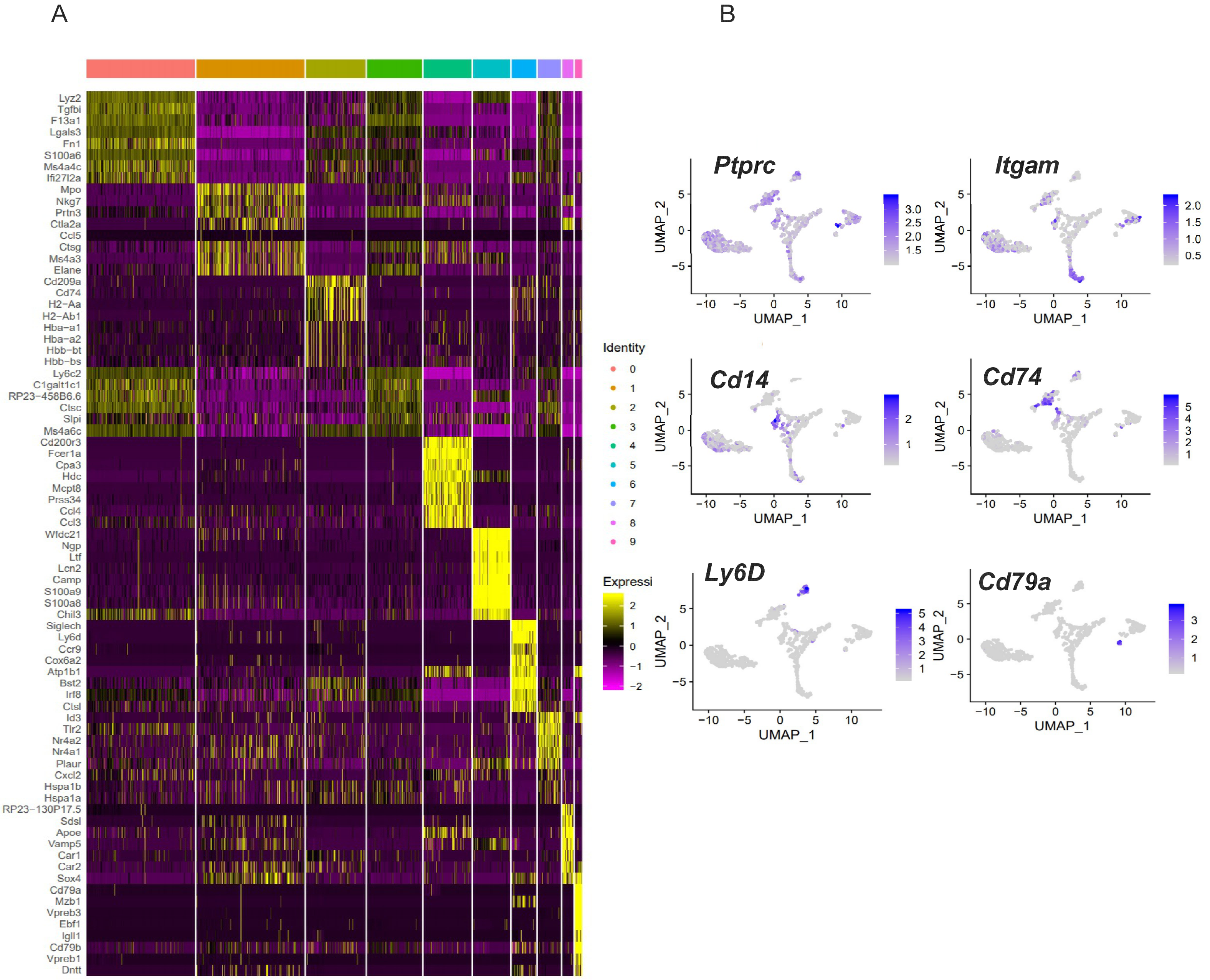
A
B
Lyz2
Tgfbi
F13a1
Lgals3
Fn1
S100a6
Ms4a4c
Ifi27l2a
Mpo
Nkg7
Prtn3
Ctla2a
Ccl5
Ctsg
Ms4a3
Elane
Cd209a
Cd74
H2−Aa
H2−Ab1
Hba−a1
Hba−a2
Hbb−bt
Hbb−bs
Ly6c2
C1galt1c1
RP23−458B6.6
Ctsc
Slpi
Ms4a6c
Cd200r3
Fcer1a
Cpa3
Hdc
Mcpt8
Prss34
Ccl4
Ccl3
Wfdc21
Ngp
Ltf
Lcn2
Camp
S100a9
S100a8
Chil3
Siglech
Ly6d
Ccr9
Cox6a2
Atp1b1
Bst2
Irf8
Ctsl
Id3
Tlr2
Nr4a2
Nr4a1
Plaur
Cxcl2
Hspa1b
Hspa1a
RP23−130P17.5
Sdsl
Apoe
Vamp5
Car1
Car2
Sox4
Cd79a
Mzb1
Vpreb3
Ebf1
Igll1
Cd79b
Vpreb1
Dntt
Identity
0
1
2
3
4
5
6
7
8
9
Expressi
2
1
0
−1
−2
Ptprc
Itgam
Cd14
Cd74
Ly6D
Cd79a
UMAP_1
UMAP_2

Figure S7

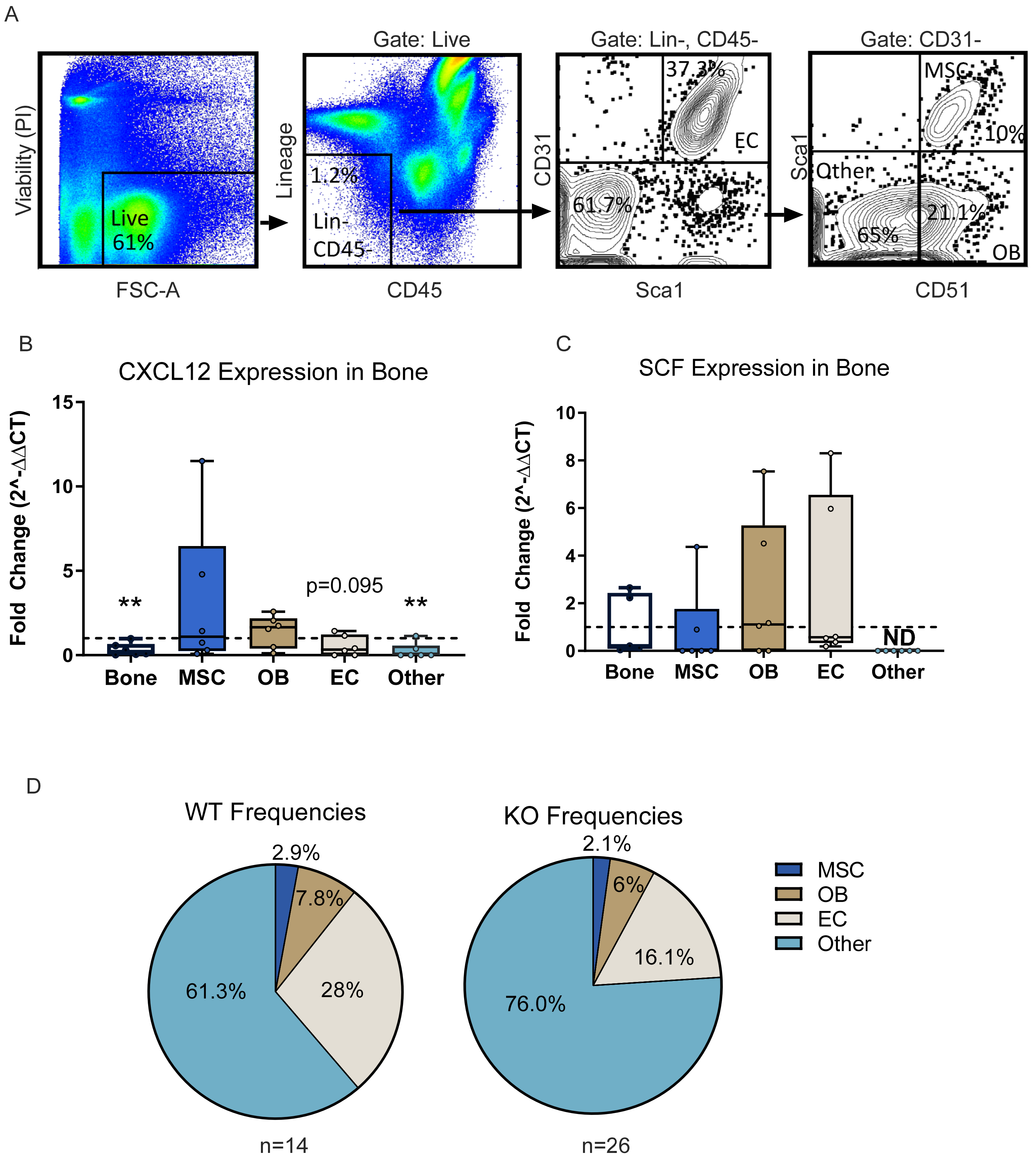
A
Gate: Live
Gate: Lin-, CD45-
Gate: CD31-
Viability (PI)
Live
61%
FSC-A
Lineage
1.2%
Lin-
CD45-
CD45
CD31
37.3%
EC
61.7%
Sca1
Sca1
MSC
10%
Other
21.1%
65%
OB
CD51
B
CXCL12 Expression in Bone
Fold Change (2^-ΔΔCT)
15
10
5
0
**
p=0.095
**
Bone
MSC
OB
EC
Other
C
SCF Expression in Bone
Fold Change (2^-ΔΔCT)
10
8
6
4
2
0
ND
Bone
MSC
OB
EC
Other
D
WT Frequencies
2.9%
7.8%
28%
61.3%
n=14
KO Frequencies
2.1%
6%
16.1%
76.0%
n=26
MSC
OB
EC
Other

Figure S8

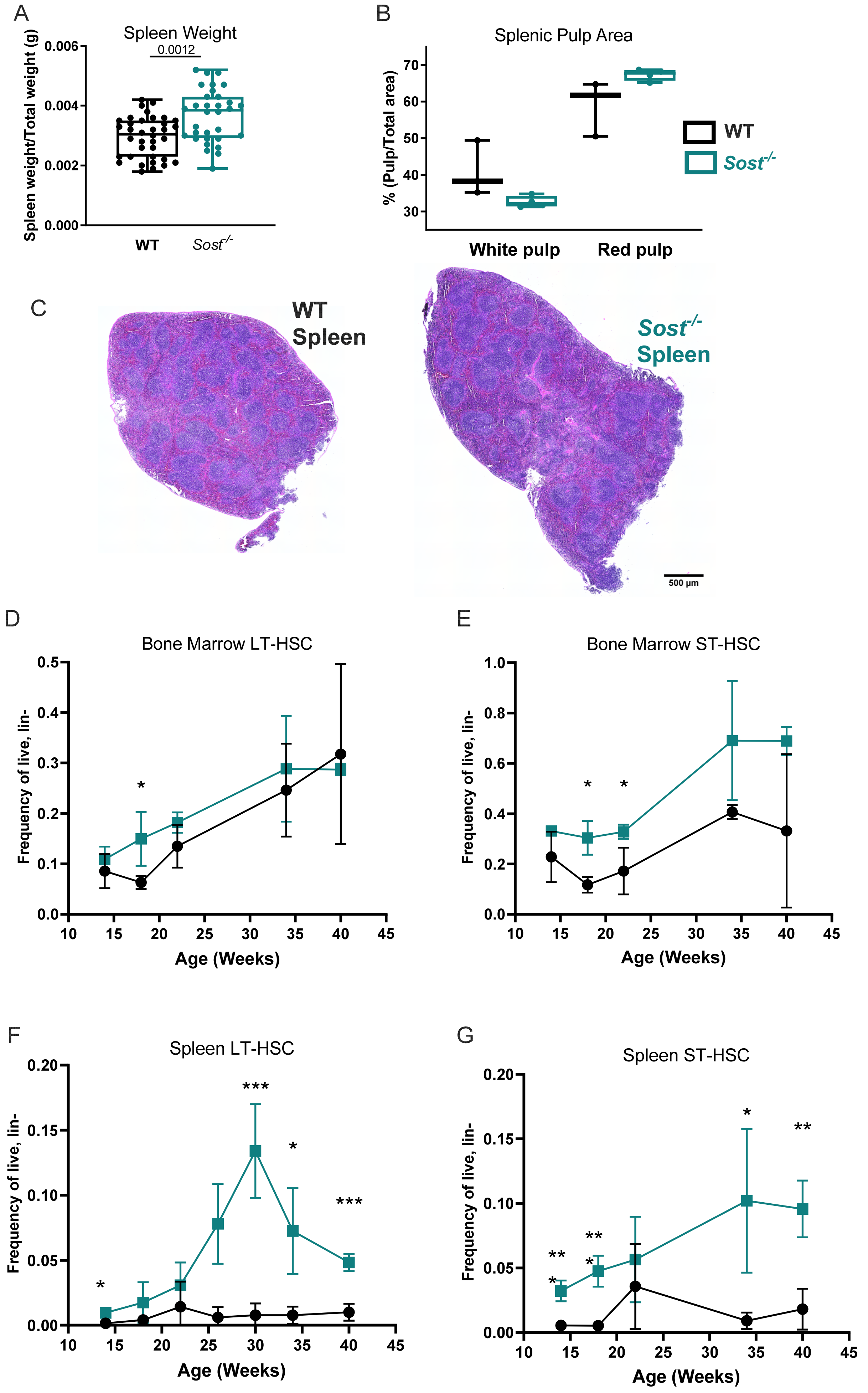
A
Spleen Weight
0.0012
Spleen weight/Total weight (g)
WT
Sost-/-
B
Splenic Pulp Area
% (Pulp/Total area)
White pulp
Red pulp
WT
Sost-/-
C
WT Spleen
Sost-/- Spleen
500 µm
D
Bone Marrow LT-HSC
Frequency of live, lin-
Age (Weeks)
E
Bone Marrow ST-HSC
Frequency of live, lin-
Age (Weeks)
F
Spleen LT-HSC
Frequency of live, lin-
Age (Weeks)
G
Spleen ST-HSC
Frequency of live, lin-
Age (Weeks)

Figure S9

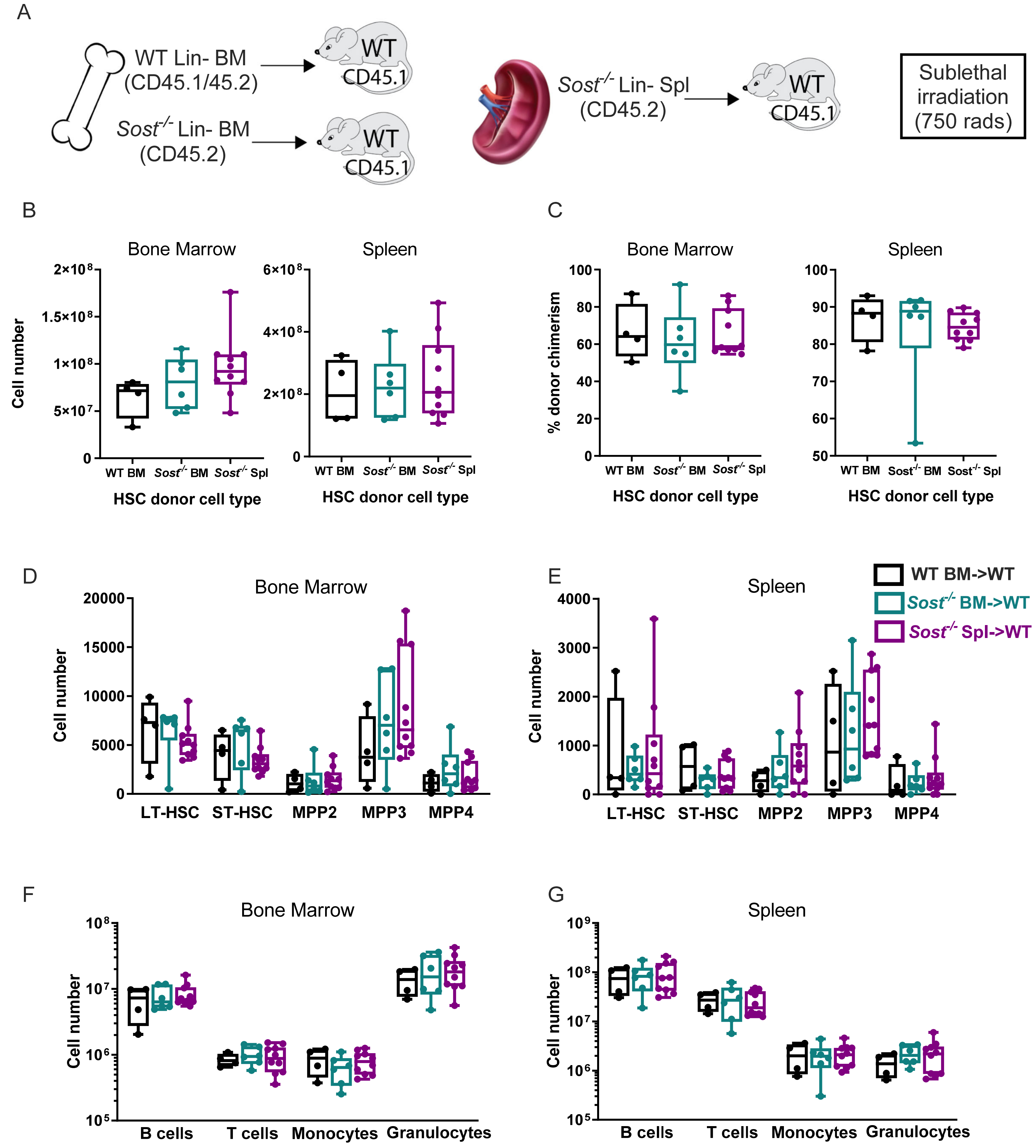
A
WT Lin- BM
(CD45.1/45.2)
WT
CD45.1
Sost-/- Lin- BM
(CD45.2)
WT
CD45.1
Sost-/- Lin- Spl
(CD45.2)
WT
CD45.1
Sublethal
irradiation
(750 rads)
B
Bone Marrow
Spleen
Cell number
HSC donor cell type
WT BM
Sost-/- BM
Sost-/- Spl
C
Bone Marrow
Spleen
% donor chimerism
HSC donor cell type
WT BM
Sost-/- BM
Sost-/- Spl
D
Bone Marrow
Cell number
LT-HSC
ST-HSC
MPP2
MPP3
MPP4
E
Spleen
Cell number
WT BM->WT
Sost-/- BM->WT
Sost-/- Spl->WT
F
Bone Marrow
Cell number
B cells
T cells
Monocytes
Granulocytes
G
Spleen
Cell number
B cells
T cells
Monocytes
Granulocytes
